# Identifying signatures of natural selection in Indian populations

**DOI:** 10.1371/journal.pone.0271767

**Published:** 2022-08-04

**Authors:** Marla Mendes, Manjari Jonnalagadda, Shantanu Ozarkar, Flávia Carolina Lima Torres, Victor Borda Pua, Christopher Kendall, Eduardo Tarazona-Santos, Esteban J. Parra

**Affiliations:** 1 Departamento de Genética, Ecologia e Evolução, Instituto de Ciências Biológicas, Universidade Federal de Minas Gerais, Belo Horizonte, MG, Brazil; 2 Department of Anthropology, University of Toronto—Mississauga Campus, Mississauga, ON, Canada; 3 Symbiosis School for Liberal Arts (SSLA), Symbiosis International University (SIU), Pune, India; 4 Department of Anthropology, Savitribai Phule Pune University, Pune, India; 5 Institute for Genome Sciences, University of Maryland School of Medicine, Baltimore, MD, United States of America; Xiamen University, CHINA

## Abstract

In this study, we present the results of a genome-wide scan for signatures of positive selection using data from four tribal groups (Kokana, Warli, Bhil, and Pawara) and two caste groups (Deshastha Brahmin and Kunbi Maratha) from West of the Maharashtra State In India, as well as two samples of South Asian ancestry from the 1KG project (Gujarati Indian from Houston, Texas and Indian Telugu from UK). We used an outlier approach based on different statistics, including PBS, xpEHH, iHS, CLR, Tajima’s D, as well as two recently developed methods: Graph-aware Retrieval of Selective Sweeps (GRoSS) and Ascertained Sequentially Markovian Coalescent (ASMC). In order to minimize the risk of false positives, we selected regions that are outliers in all the samples included in the study using more than one method. We identified putative selection signals in 107 regions encompassing 434 genes. Many of the regions overlap with only one gene. The signals observed using microarray-based data are very consistent with our analyses using high-coverage sequencing data, as well as those identified with a novel coalescence-based method (ASMC). Importantly, at least 24 of these genomic regions have been identified in previous selection scans in South Asian populations or in other population groups. Our study highlights genomic regions that may have played a role in the adaptation of anatomically modern humans to novel environmental conditions after the out of Africa migration.

## Introduction

South Asia was one of the first geographic areas colonized during the out-of-Africa migration of anatomically modern humans, and not surprisingly, is characterized by having one of the highest levels of genetic diversity outside of Africa [1–5]. This diversity has been shaped by different evolutionary and demographic factors including bottlenecks and genetic drift, multiple migration waves, endogamy and natural selection [6–13]. Recent studies have highlighted three important migration events that contributed to the formation of present-day South Asian populations. Briefly, these three major events correspond to the I) original out of Africa migration that eventually gave rise to the *Ancient Ancestral South Indian* population (AASI), II) the migration of Neolithic farmers, primarily from the Iranian plateau, and III) the Bronze Age migration of the Yamnaya Steppe Pastoralists [14–17]. It has been proposed that an initial admixture process between AASI hunter-gatherers and Iranian-related farmers gave rise to a population that has been named the *Indus Periphery* group. Further admixture events of the *Indus Periphery* populations with AASI southeastern groups and northwestern groups with Steppe ancestry gave rise to the *Ancestral South Indian* (ASI) and *Ancestral North Indian* (ANI) populations, respectively, a process that probably occurred in the second millennium BCE [17]. Most of the modern human populations in South Asia show varying proportions of ASI and ANI ancestry [10, 12, 17, 18]. It is important to note that there have been many other documented demographic events in South Asia, including invasions from the Greeks, Kushans, Huns, Muslims, Moghuls, and the English [19].

In the context of the complex organization of the Indian society, some classifications have been attempted, so for the purpose of this study, we will use the division of Modern Indians into tribal and non-tribal groups. Tribal groups are considered the Indigenous populations, while non-tribal groups comprise social hierarchical endogamous castes as well as the religious groups outside the caste system [20]. In general, tribal populations have a smaller population size than caste populations and consequently have experienced more intensively the effects of genetic drift [13, 21].Genetic studies have indicated that the shift to both endogamous and consanguineous marriages, which is characteristic of the caste system in India, occurred around 2,000–1,500 years ago, as there is evidence of substantial admixture in this region prior to this time [9, 10, 17, 18, 20, 22, 23].

In contrast with the recent advances in our understanding of the demographic history of the South Asian continent [9, 13, 16–18, 24–27], there have been limited attempts to explore the potential role of positive natural selection on South Asian populations. As humans migrated out-of-Africa, they adapted to novel environments and it is of interest to identify the genomic regions that were targeted for positive natural selection. There have been many efforts to identify selection signatures in European, East Asian and African populations [28–31], but just a few studies have focused on South Asian samples [6, 24, 32, 33].

Here, we present the results of a genome-wide scan for signatures of positive selection using data from four tribal groups (Kokana, Warli, Bhil, and Pawara) and two caste groups (Deshastha Brahmin and Kunbi Maratha) from West Maharashtra, as well as two samples of South Asian ancestry from the 1000 Genome Project (Gujarati Indian from Houston, Texas and Indian Telugu from UK) (S1 Fig). In order to identify putative genomic regions under positive selection, we used tests of positive selection based on different statistics, including Population Branch Statistic (PBS), Cross-population Extended Haplotype Homozygosity (xpEHH), Integrated Haplotype Score (iHS), Composite Likelihood Ratio (CLR), Tajima’s D, as well as two recently developed methods: Graph-aware Retrieval of Selective Sweeps (GRoSS)—that uses admixture graphs to infer signatures of selection in specific branches of the graphs [34] and Ascertained Sequentially Markovian Coalescent (ASMC)—a coalescence-based method [35].

## Materials and methods

### Datasets

In this study, we used two different datasets to enable a deeper understanding of positive natural selection signatures in Indian populations. The first dataset is genome-wide data from six West Maharashtra (WM) populations, belonging to the Indo-European language family (S1 Fig). Those populations include four tribal populations (collected from Jawhar at 19.918N, 73.238E and Dhadgaon at 21.828N, 74.228E): Kokana, Warli, Bhil, and Pawara; and two caste groups (collected close to Pune city at 18.538N, 73.878E): Deshastha Brahmins, and Kunbi Marathas [36] (S1 Fig). The sampled individuals are 480 volunteers who provided informed written consent, administered in local vernacular, and information about their place of origin, clan, age, and gender along with 5–8ml of whole blood, collected in EDTA vials. The project was approved by the Institutional Ethics Committee (IEC) at the Savitribai Phule Pune University (Ethics/2012/16). All subjects were explained the nature and objectives of the study orally and were given an information sheet in Marathi with details of the study. The researcher and university details were mentioned in case they had additional questions. Talk/orientation sessions were also held with study participants and the general public (e.g., community members) to explain the details of the study. DNA extraction was performed using the phenol-chloroform method [37] and DNA was concentration was quantified with an Eppendorf BioPhotometer plus. Genotyping was carried out with Applied Biosystem’s Axiom TM Precision Medicine Research Array (PMRA) at Imperial Life Sciences Pvt Ltd. Laboratory (Gurgaon, Haryana, India) using standard protocols. This array includes 902,981 genetic markers.

The second dataset corresponds to the 1KGP Phase 3 data [38] from Indian ancestry: GIH (Gujarati Indian from Houston, Texas), and ITU (Indian Telugu from UK); the European CEU sample (Utah Residents (CEPH) with Northern and Western European Ancestry); and the African YRI sample (Yoruba in Ibadan, Nigeria) samples. We used this dataset in two ways: 1) To carry out diverse tests of selection based on the SNPs that overlap with the microarray-based sample from West Maharashtra described above, and 2) To carry out diverse tests of selection based only on the high coverage 1000 genome data (~70,7M autosomal SNPs) to validate the previous results [39].

### Quality control

The first QC in the WM samples was done with the Axiom Analysis Suite program which retained ~522,125 polymorphic markers and 478 samples [40].

We did additional QC steps to remove samples based on: 1) sex discrepancies, 2) outliers for heterozygosity, 3) missing call rates <0.95, 4) related individuals (pi-hat> 0.25), and 5) samples that were outliers in Principal Component Analysis (PCA) plots. We also removed markers with: 1) genotype call rate <0.95, 2) Hardy-Weinberg (HW) p-values <10–6, 3) minor allele count <4, 4) Insertion/Deletion (Indel) markers, 5) markers not present in the 1000 Genomes reference panel, or that did not match the chromosome, position, or alleles information, 6) A/T or G/C SNPs, 7) allele frequency differences > 20% between the study sample and the 1000 Genomes South Asian reference sample, 8) SNPs without chromosome information and 9) duplicated SNPs. After all QC steps, the dataset contained ~365,152 variants and 456 samples. Similar QC steps were carried out in the 1KG sample, obtaining a final sample with ~54.2 Milions variants.

### Natural selection analysis

Our approach to identify putative regions of positive selection is based on the application of several methods that focus on different aspects of the genomic data in order to identify “outlier” regions based on the empirical distribution of test statistics across the genome. We carried out an initial scan based on the SNPs that overlap between the WM and the 1KG samples (~283K SNPs) using six different approaches: PBS, xpEHH, iHS, CLR, Tajima’s D and GRoSS (S2 Fig).

We selected the top 1% regions for each method (the greater values for PBS, xpEHH, iHS, CLR and the lowest values for Tajima’s D and GRoSS P-values). We then annotated these genomic regions using the UCSC (University of California Santa Cruz) Table Browser tool (https://genome.ucsc.edu/), which searches for the specific genes found in each genomic region. In our tables and figures, we provide additional details on the outlier regions (top 1%, top 0.5% and top 0.1%).

**Fig 1:**
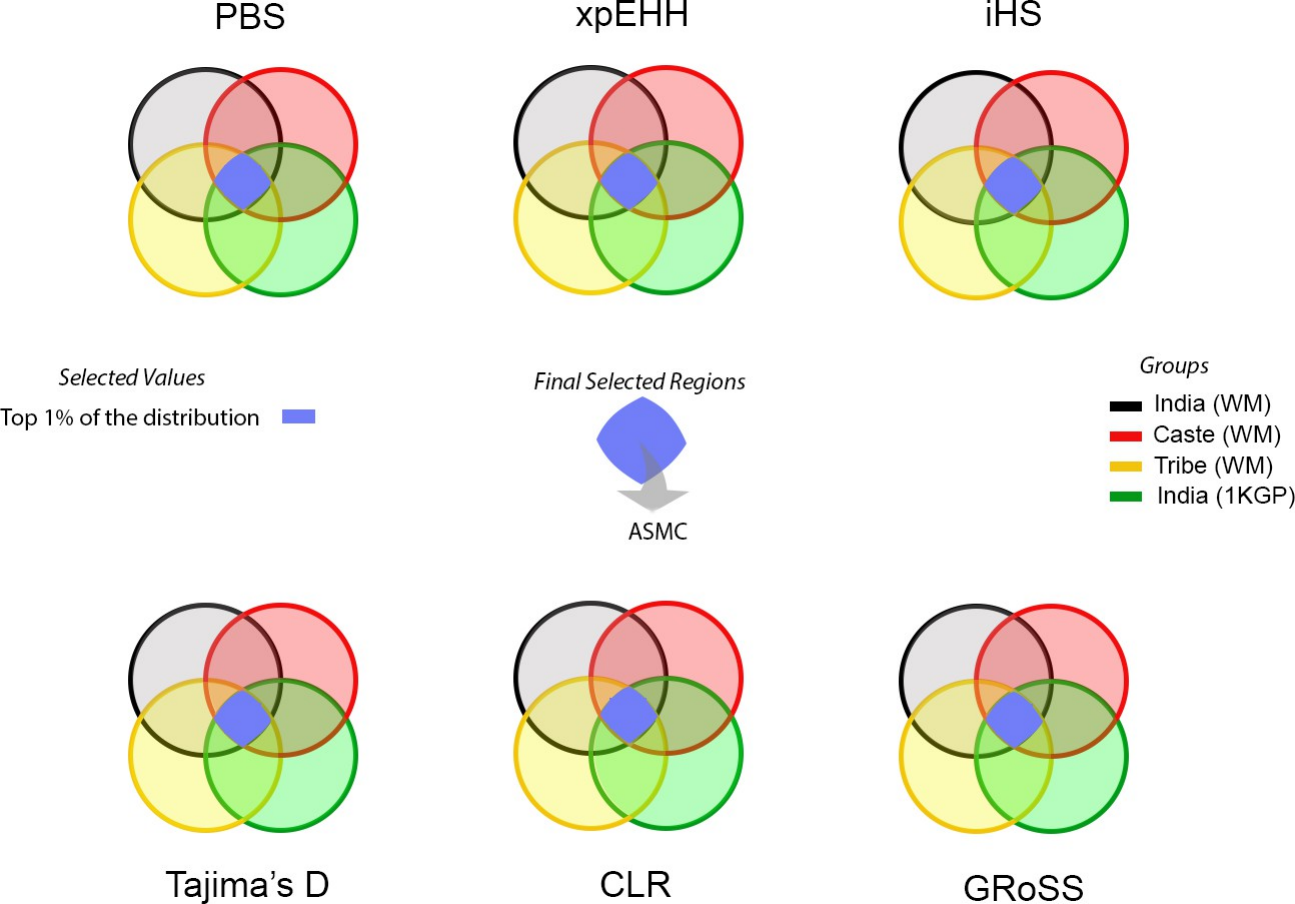
Schematic representation of the approach to identify putative selective regions. We applied six different methods to identify outliers (top 1% results) and selected regions that were observed in all population groups and were outliers for at least two independent methods. Additionally, we performed analyses using a novel coalescence-based method implemented in the program ASMC.

In order to minimize false-positive results, as an additional step, we filtered the results using two strategies: 1/ We only selected genomic regions that were pointed out for any given method in the four samples of India included in our study (WM-Castes, WM-Tribes, WM-All and 1KG-India and 2/ We only selected genomic regions that are outliers for at least two different methods (Fig 1). We also evaluated if the regions identified in these analyses show unusual coalescence patterns in Indian populations using the recently developed ASMC method [35]. Finally, we also evaluated the results of the selection tests based only on the high coverage 1KG Indian samples as a strategy to validate the results.

### Methods based on population differentiation

#### PBS

To identify changes in the allele frequencies of a target population since its divergence from an ancestral population we performed a PBS test. This statistic is based on the comparison of the allele frequency differences measured with F_ST_ values among three groups: 1) a target population; 2) a sister population, and 3) an outgroup [41]. This method can identify signatures of natural selection mainly between 75K and 50K years ago and is sensitive both to positive natural selection acting on standing variation or on *de novo* mutation [42].

We applied a MAF filter (Minimum Allele Frequency > 0.05) where ~82,291 variants were removed. The F_ST_ values were computed using 4P software [43] and the PBS formula was applied as follows [41]:

$$\text{PBS}=\left({\text{F}}_{\text{ST}}\text{T}1+{\text{F}}_{\text{ST}}\text{T}2-{\text{F}}_{\text{ST}}\text{T}3\right)/2$$

Where the F_ST_T values correspond to the F_ST_ computed with 4P and transformed according to [44]:

$${\text{F}}_{\text{ST}}\text{T}=-\text{log}\left(1-{\text{F}}_{\text{ST}}\right)$$

Therefore:

F_ST_T1: transformed F_ST_ between the target population and the sister population.
F_ST_T2: transformed F_ST_ between the target population and the outgroup.
F_ST_T3: transformed F_ST_ between the sister population and the outgroup.

The PBS values were normalized following the formula [45]:

$$\text{PBSn}=\text{PBS}1\;/\;\left(1+\text{PBS}1+\text{PBS}2+\text{PBS}3\right)$$

Where:

PBSn: normalized PBS.
PBS1: estimated PBS when the PBS is calculated for the target population.
PBS2: estimated PBS when PBS is focused on the sister population.
PBS3: estimated PBS when PBS is focused on the outgroup.

In all PBS analyses, we used the CEU as the sister population and YRI as the outgroup. We performed the test for each of the following target populations: 1) India WM, 2) Caste WM, 3) Tribe WM, and 4) India 1KGP populations together (ITU and GIH). We identified putatively selected regions showing extreme PBS values using *in-house* scripts. For these analyses, we used bins of 20 SNPs with 5 SNPs of overlap.

#### GRoSS

To incorporate the genetic history of the Indian populations in the inference of natural selection events, we applied the Graph-aware Retrieval of Selective Sweeps (GRoSS) software [34]. This method uses complex admixture graphs to infer signatures of natural selection along the branch of the graph, based on the statistics developed by Racimo et al, 2018 [46]. In order to detect polygenic adaptation in admixture graphs, GRoSS uses data frequency information and the population history graph topology. Based on the patterns of allele frequency in the populations in the admixture graph, the method estimates a P-value for each target polymorphic site. Low P-values correspond to strong deviations from neutrality along a particular branch of the graph.

To infer the admixture graph we use qpGraph [10] with two configurations (S3 Fig): one with the final leaf being the Indian population (S3A Fig) and the other with the final leaves being Tribe and Caste groups (S3B Fig). Then, we run GRoSS with the output “.dot” from qpGraph and the data formatted in “.gross” from 1) India (WM), 2) Caste (WM) and Tribe (WM) and 3) India 1kgp.

To analyze the GRoSS results, we focus on the branch between Europe2 and the target population, India (WM), India 1kgp (S3A Fig), Caste (WM), Tribe (WM) (S3B Fig).

Regions showing a strong deviation of neutrality will have low P-values [34]. We annotated the top genomic regions identified with GRoSS by creating windows spanning 100Kb before and after the SNP with the lowest P-value for those regions.

### Methods based on linked variation

This group of methods focuses on more recent *de novo* mutations and is particularly powered to identify selective events that happened approximately less than 30,000 years ago [42, 47]. To cover this time span, we apply xpEHH [48] and iHS [49]. The principle of those methods is based on the fact that a positive selection event increases the frequency of a variant and of the variants close to it, faster than the recombination or mutation process breaks those haplotypes, generating a high-frequency long-range haplotype [48].

The xpEHH method incorporates the calculation of the EHH for all SNPs in 1MB of distance forwards and backwards for two target populations, in this case, India and CEU [48], while iHS tracks the decay of homozygosity in the target haplotype concerning to the ancestral and derived haplotypes extending from a specific site [50].

For both analyses we apply the software Selscan [50] with default parameters, in our data, phased with Sanger Imputation Service, using EAGLE2 [51] for our samples data (West Maharashtra) and Shapeit4 for the 1KGP High Coverage data [52] using the GRCh37 genetic map and the MCMC parameters:–mcmc-iterations 10b,1p,1b,1p,1b,1p,1b,1p,10m, which perform 10 burn-in iterations, followed by four paired runs of pruning and burn-in, and, finally, 10 main iterations of sampling. Both results were normalized with the extension “norm” from Selscan, by 20 equally sized allele frequency bins. In the iHS inference, we used polarized data. After identifying the SNPs with the highest values for iHS and xpEHH, we annotated the genomic regions by creating windows spanning 100 Kb before and after the selected SNPs.

### Methods based on site frequency spectrum

These methods can detect older natural selection events, ~80,000 years ago, and although Tajima’sD is used mostly for sequence data, we apply it in this study combined with other methods, to identify regions whose frequency spectra are strongly different from the bulk of the genome, suggesting the influence of selection [47, 49].

This test compares the average number of pairwise differences and the average number of total segregating sites [53]. Strong negative Tajima’sD values suggest an excess of rare alleles, which may be indicative of positive selection or population expansion [54]. To estimate Tajima’s D, we used vcftools with the “TajimaD” flag, for 100 kb windows [55], and identified the regions with the most negative values as regions under putative selective pressure.

### Composite likelihood ratio method

To reduce the ratio of false positives, this approach combines test scores from diverse sites across a contiguous region [54]. In this study, we computed the Composite likelihood ratio (CLR) [56] with SweeD, which calculates this test using the relation between the likelihood of a sweep at a certain position in the genome by the product of the empirical site frequency spectrum over all SNPs [57]. We ran SweeD with the phased and polarized data, in a resolution of 200Kb windows by chromosome, which corresponds to an average of 20 SNPs by each window, but we just consider the windows with more than 10 SNPs. We considered the higher values as an indicator of positive selection.

### ASMC

The rapid rise in frequency of a beneficial allele due to a recent positive natural selection event provokes the coalescence of all individuals with the beneficial allele to a more recent common ancestor than expected under a neutral model. Thus, we checked if our results showed an unusually high density of very recent inferred Time to the Most Common Recent Ancestor (TMRCA) events using the Ascertained Sequentially Markovian Coalescent (ASMC) method [35]. To run ASMC we followed the steps recommended by the authors. Then, we merged, normalized and plotted our results.

## Results

To achieve our aim to detect putative signatures of natural selection in the South Asian population with the minimum of false-positive results, we applied six different approaches in a dataset including ~283K SNPs markers that overlap between the WM and 1KG-India samples (Fig 1) and we report genomic regions that were identified as outliers with at least two independent methods in all the samples analyzed.

**Fig 2:**
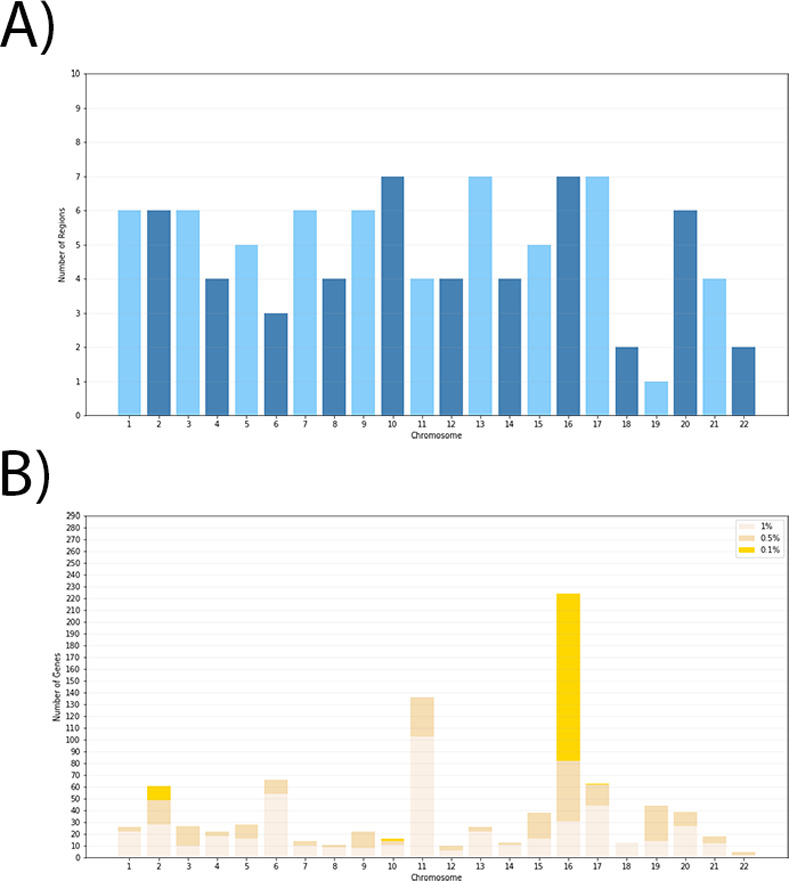
Overview of our results. A) Distribution of the number of regions identified for each chromosome; B) Distribution of the number of genes located within putative selective regions identified with two or three methods for each chromosome, for all thresholds (1%, 0.5% and 0.1%).

Our analysis identified a total of 107 genomic regions overlapping 433 genes distributed across all autosomal chromosomes, (Fig 2 - shows the number of regions (Fig 2A), and the number of genes in the top 1%, 0.5% and 0.1% of the distribution (Fig 2B). S1 Table reports these signals, including information about the methods for which the regions were identified as outliers. These regions are also reported in a condensed form in S2 Table). Next, we compared the results identified using these six methods with those based on a recently described coalescence-based approach implemented in the package ASMC [35]. The graphs with the results corresponding to this method are presented for each chromosome as supplementary material (S4 Fig). We observed considerable congruence between our original signals and the results obtained with ASMC. Many of the putative genomic regions identified in our initial scan also show recent inferred times to the most recent common ancestor (TMRCA) using ASMC, as expected under recent positive selection. The ASMC method tends to show higher resolution than the other methods, with narrower regions that include a smaller number of genes.

Chromosome 16 is particularly rich in the number of genes within regions putatively under selection (Fig 2B), primarily due to a single region of 2.6 Mb on chromosome 16 that alone contains 78 genes. This region also shows enrichment in recent inferred TMRCA events using the ASMC program (S5 Fig).

We analyzed for each method what is the proportion of signals that are shared by each pair of populations and the proportion of signals that are shared by different methods. This is presented in graphical format in S6 and S7 Figs.

There have been previous efforts to identify signatures of selection using exclusively South Asian samples [6, 24, 32, 33], or including South Asian samples in the analyses [7]. In Table 1, we highlight the genomic regions identified in our study that have been also reported in previous studies.

**Table 1:** List of the putative selected genomic regions that have been described in previous studies.

chr	Start	End	SNPs	genes	OBS	Shared signal with 1KGP_HC	Shared signal with PopHuman Browser (iHS)
1	234663636	235491532	118	LOC100506795,TOMM20,SNORA14B,RBM34,ARID4B,MIR4753	**Also reported on Karlsson et al. 2013**	xpEHH(0.5%),PBS(0.1%)	GIH,ITU
2	72356366	73053177	20	CYP26B1,EXOC6B,SNORD78	**Also reported on Liu et al. 2017, Also reported on Karlsson et al. 2013**	xpEHH(0.5%), Tajimas`D(0.5%), PBS(0.5%)	GIH,ITU
2	96940073	98858761	56	SNRNP200,ITPRIPL1,NCAPH,ARID5A,KANSL3,FER1L5,ANKRD39,SEMA4C,FAM178B,FAHD2B,ANKRD36,ANKRD36B,COX5B,ACTR1B,LOC728537,ZAP70,VWA3B	**Also reported on Liu et al. 2017, Also reported on Karlsson et al. 2013**	PBS(0.1%), xpEHH(0.1%)	GIH,ITU
2	241662829	242033643	60	KIF1A,AGXT,C2orf54,SNED1	**Also reported on Karlsson et al. 2013**	xpEHH(1%)	GIH,ITU
4	39289068	39529218	26	RFC1,KLB,RPL9,LIAS,LOC401127,UGDH	**Also reported on Karlsson et al. 2013**	xpEHH(0.5%)	GIH,ITU
6	29550028	33086926	3545	SNORD32B,OR2H2,GABBR1,MOG,ZFP57,HLA-F,HLA-F-AS1,IER3,AK098012,DDR1,MIR4640,GTF2H4,VARS2,MUC22,HLA-C,HLA-B,HCP5,PMSP,PRRT1,LOC100507547,PPT2,PPT2-EGFL8,EGFL8,AGPAT1,RNF5,AGER,PBX2,GPSM3,NOTCH4,HLA-DMB,HLA-DMA,BRD2,HLA-DOA,HLA-DPA1,HLA-DPB1,HLA-DPB2	**Also reported on Liu et al. 2017 Also reported on Suo et al. 2012**	PBS(0.5%), xpEHH(1%)	GIH,ITU
7	111366163	111461829	12	DOCK4,BC043243	**Also reported on Karlsson et al. 2013**	xpEHH(0.5%), PBS(0.5%)	GIH, ITU
7	119913721	120390387	19	KCND2	**Also reported on Liu et al. 2017, Also reported on Karlsson et al. 2013**	xpEHH(0.5%), Tajimas`D(0.5%), CLR(0.5%)	GIH, ITU
9	123714613	124095120	18	C5,CNTRL,RAB14,GSN	**Also reported on Metspalu et al. 2011, Also reported on Karlsson et al. 2013**	xpEHH(0.5%), PBS(0.1%)	GIH,ITU
10	320129	735608	23	DIP2C	**Also reported on Karlsson et al. 2013**	xpEHH(0.5%), Tajimas`D(1%)	GIH,ITU
10	118187423	118261387	9	PNLIPRP3,JA611286	**Also reported on Karlsson et al. 2013**	Tajimas`D(0.5%)	
11	61447904	62622555	144	DAGLA,MYRF,DKFZP434K028,BC020196,TMEM258,MIR611,FEN1,FADS1,MIR1908,FADS2,FADS3,RAB3IL1,BEST1,FTH1,BC132896,SNORD27	**Also reported on Suo et al. 2012**	PBS(0.1%), xpEHH(0.1%)	GIH, ITU
11	65479472	68846261	198	KAT5,RNASEH2C,AP5B1,SNX32,CFL1,MUS81,EFEMP2,CTSW,FIBP,CCDC85B,FOSL1,KLC2,RAB1B,AK125412,CNIH2,YIF1A,TMEM151A,CD248,RIN1,BRMS1,B3GNT1,SLC29A2,AX747485,NPAS4,MRPL11,LOC100130987,POLD4,CLCF1,RAD9A,PPP1CA,TBC1D10C,CARNS1,RPS6KB2,PTPRCAP,CORO1B,GPR152,CABP4,TMEM134,AIP,PITPNM1,CDK2AP2,CABP2,C11orf24,LRP5,MRGPRF,BC039516,TPCN2	**Also reported on Karlsson et al. 2013**	CLR(0.1%),Tajimas`D(0.5%), xpEHH(0.1%), PBS(0.5%)	GIH, ITU
11	126293395	132206716	884	KIRREL3,DJ031150,NTM	**Also reported on Metspalu et al. 2011**	xpEHH(0.5%), PBS(0.5%)	GIH,ITU
13	92050934	93519487	139	GPC5	**Also reported on Karlsson et al. 2013**	xpEHH(0.1%), PBS(1%)	GIH,ITU
14	63173944	63511955	35	KCNH5	**Also reported on Metspalu et al. 2011, Also reported on Karlsson et al. 2013**	xpEHH(0.1%), PBS(0.5%)	GIH,ITU
16	29464909	32077476	122	BOLA2,KIF22,MAZ,AB209061,AK097472,PRRT2,PAGR1,BC029255,MVP,CDIPT,CDIPT-AS1,SEZ6L2,ASPHD1,KCTD13,TMEM219,TAOK2,HIRIP3,LOC595101,CD2BP2,TBC1D10B,MYLPF,SEPT1,ZNF48,SEPT2,ZNF771,DCTPP1,SEPHS2,ITGAL,MIR4518,ZNF768,ZNF747,AK056973,ZNF764,ZNF688,ZNF785,ZNF689,PRR14,FBRS,LOC730183,SRCAP,SNORA30,LOC100862671,PHKG2,C16orf93,RNF40,ZNF629,BCL7C,MIR4519,BC073928,MIR762,CTF1,FBXL19-AS1,FBXL19,ORAI3,SETD1A,HSD3B7,STX1B,STX4,BC039500,ZNF668,ZNF646,PRSS53,VKORC1,BCKDK,KAT8,PRSS8,PRSS36,FUS,TLS/FUS-ERG,PYCARDC,16orf98,TRIM72,PYDC1,ITGAM,DL489986,ITGAX,IGHV 3–07,IGH	**Also reported on Perdomo-Sabogal et al, 2019 for Chinese in Bejing (CHB), Also reported on Karlsson et al. 2013**	Tajimas`D(0.1%), PBS(0.1%), xpEHH(0.1%)	GIH,ITU
16	46760587	47735434	15	MYLK3,C16orf87,GPT2,ITFG1,PHKB	**Also reported on Karlsson et al. 2013**	Tajimas`D(0.5%), PBS(0.5%)	
16	87117167	87457487	57	AK125749,C16orf95,FBXO31,MAP1LC3B,ZCCHC14	**Also reported on Karlsson et al. 2013**	xpEHH(0.5%), PBS(0.1%)	GIH,ITU
17	17876126	18011299	4	LRRC48,ATPAF2,BC150162,GID4,DRG2	**Also reported on Karlsson et al. 2013**	PBS(0.5%)	GIH,ITU
17	58755212	59470192	58	BCAS3	**Also reported on Metspalu et al. 2011, Also reported on Karlsson et al. 2013**	Tajimas’D(0.1%), CLR(0.1%), PBS(1%)	GIH, ITU
20	53092265	53267710	13	DOK5	**Also reported on Metspalu et al, 2011**	xpEHH(0.5%), Tajimas`D(0.1%), CLR(0.1%), PBS(0.1%)	
22	35462129	35483380	3	ISX	**Also reported on Metspalu et al, 2011**		
22	46756730	46933067	49	CELSR1	**Also reported on Metspalu et al, 2011, Also reported on Karlsson et al. 2013**	xpEHH(0.5%), Tajimas`D(0.5%)	

List of the regions with putative signatures of natural selection in our study that have been described in other studies, with a particular emphasis in studies in South Asian populations or other Asian groups. 1: Metspalu et al. 2011, 2: Suo et al. 2012, 3: Karlsson et al. 2013, 4: Liu et al. 2017, 5: Perdomo-Sabogal and Nowick, 2019. In Blue, we show regions with results in the 0.5% of the most significant values for at least one method, and in red the results in the top 0.1% most significant results for at least one method. We also highlight the regions that also have significant results in the 1kgp high coverage data (1kgp_HC), and in the PopHuman Browser with iHS.

## Discussion

Genome-wide scans for signatures of selection can provide very useful insights about the role of natural selection driving the adaptation of our species after the out of Africa migration of anatomically modern humans. In this context, very few studies have specifically focused on South Asian populations [6, 24, 32, 33], which have some of the highest genetic diversity observed outside Africa [1–5, 21].

In this study we have carried out genome-wide scans to identify putative signatures of natural selection in South Asia, using microarray-based data from several tribal and caste groups of West Maharashtra, India as well as high coverage sequencing data available from the 1KGP samples from India (GIH and ITU). We applied methods based on different strategies, including haplotype length, population differentiation, site frequency spectrum, as well as recently developed methods based on admixture graphs and locus-specific pairwise coalescence times. In order to minimize the risk of false-positive signals, we only selected regions that were identified by at least two independent methods and were present in all the samples analyzed in the study (Fig 1).

Based on these analyses, we identified 107 genomic regions comprising 433 genes as potential candidates of positive selection in Indian populations. The average number of genes per region is 4.08, and the median is 1. The results are presented in S1 and S2 Tables. To our knowledge, at least 24 of the 107 regions identified in our study have been reported before in previous efforts to identify putative signals of natural selection in South Asian and East Asian populations [6, 7, 29, 31, 32]. These regions are reported in Table 1.

We did not observe any systematic excess of sharing of signals between the two groups of West Maharashtra (tribes and castes) with respect to the comparisons of these groups with the 1KG Indian samples (S6 Fig), which may be reflective of the varied evolutionary and demographic events witnessed by these West Maharashtra caste and tribal groups. It is important to note that in a previous study [13] we showed that in Principal Component plots, the WM-Tribes and WM-Castes clustered separately from each other, whereas the WH-Castes were located closer to the 1KG South Asian samples. Additionally, Identity-By-Descent (IBD) analyses indicated that the WM-Tribes have had smaller effective population sizes and have been under stronger influence of genetic drift than the WM-Castes.

When comparing the results for each method in the different groups (WM-Castes, WH- Tribes, WM-All and India-1KG, S6 Fig), we observed some variation in the percentage of shared signals depending on the method. Previous studies have also reported limited overlap between different approaches used to identify signatures of selection [8, 58]. This is not surprising given that these methods are based on different characteristics of the data (e.g., population differentiation, linked variation, site frequency spectrum) and have different sensitivities to detect selective events depending on factors such as time and type of selection [59, 60]. Our strategy has been to select outliers identified with more than one method in all population groups in order to more reliably identify putative genomic regions under selection.

Additionally, we analyzed the data using a recently developed method based on locus-specific pairwise coalescence times (ASMC), and observed that in general this method shows concordant results with respect to those observed based on the other approaches, and in some cases provides a higher level of resolution. S4 Fig shows the graphs generated by the program ASMC for each chromosome, including additional information about some specific regions (e.g. gene information and overlap with results described in previous studies). The genomic regions with the highest enrichment of recent coalescence events in our ASMC analysis (higher than 7) were located on chromosomes 1 (~248Mb), 4 (~80Mb), 6 (~29.5Mb), and 16 (~1Mb). The genes overlapping these regions are highlighted in S4 Fig.

We observed a substantial overlap of our signals with those reported in a natural selection study using samples of Bengali ethnicity from Bangladesh (BEB) that applied the Composite of Multiple Signals (CMS) method [32]. This study explored the relationship of selection signals with selective pressure due to cholera. The authors reported that a number of genes identified in the putative selected regions were associated with cholera susceptibility in two separate cohorts. The region with the strongest signal of selection, located on chromosome 2 and encompassing five genes (*NRNP200*, *CIAO1*, *ITPRIPL1*, *NCAPH*, and *TMEM127)* was also the region showing the strongest association with cholera, with the top associated SNPs located between the genes *NRNP200* and *ITPRIPL1*. We identified a very large region on chromosome 2 that spans almost 2 Megabases (from 96.9 Mb to 98.8 Mb) showing signatures of selection, which includes these two genes (Tables 1 and S1 and S2). It is important to note that Karlsson et al. (2013) described three independent putative selected regions in this genomic interval, the first from positions 96.2 to 96.4 Mb (including the *NRNP200* and *ITPRIPL1* genes), the second from positions 97.5 to 97.7 Mb (including *COX5B*, *ACTR1B* and *ZAP70*), and the third from positions 98.1 to 98.4 Mb (including *VWA3B* and *CNGA3*). All of these genes were identified in our initial analysis, and this broad region also shows the largest ASMC enrichment on chromosome 2 (S8 Fig). The region from chromosome 2 from 97.1 to 98.4 was also identified by Liu et al. (2017) [7] in three samples from South Asia. In summary, there is strong evidence of positive selection acting on this genomic region in South Asian populations, but further studies will be required to elucidate the specific target/s of selection and the selective factors involved. Karlsson et al. (2013) [32] also reported associations with cholera in three additional putative selected regions when focusing on the most severe cholera cases, encompassing the potassium ion transport genes *KCNH7* and *KCNH5*, and the ribosomal protein kinase gene *RPS6KB2*. Two of these three genes (*KCNH5* and *RPS6KB2*) were also identified in our study. In our analysis, *KCNH5* was the only gene present in a relatively narrow interval on chromosome 14 from 63.1 to 63.5 Mb (Table 1). A broader region on chromosome 14 (from 61.6 Mb to 64 Mb) including *KCNH5* was also identified as a putative selective signal in a study by Metspalu et al. (2011) [6] in South Asian samples and this region shows strong enrichment in our ASMC results (S4N Fig). In contrast, in our analyses, *RPS6KB2* is one of many genes identified in a very broad region on chromosome 11 spanning more than 3 Mb (from 65.4 to 68.8 Mb, Table 1). This region also shows strong enrichment in our ASMC analyses (S4K Fig).

**Fig 3:**
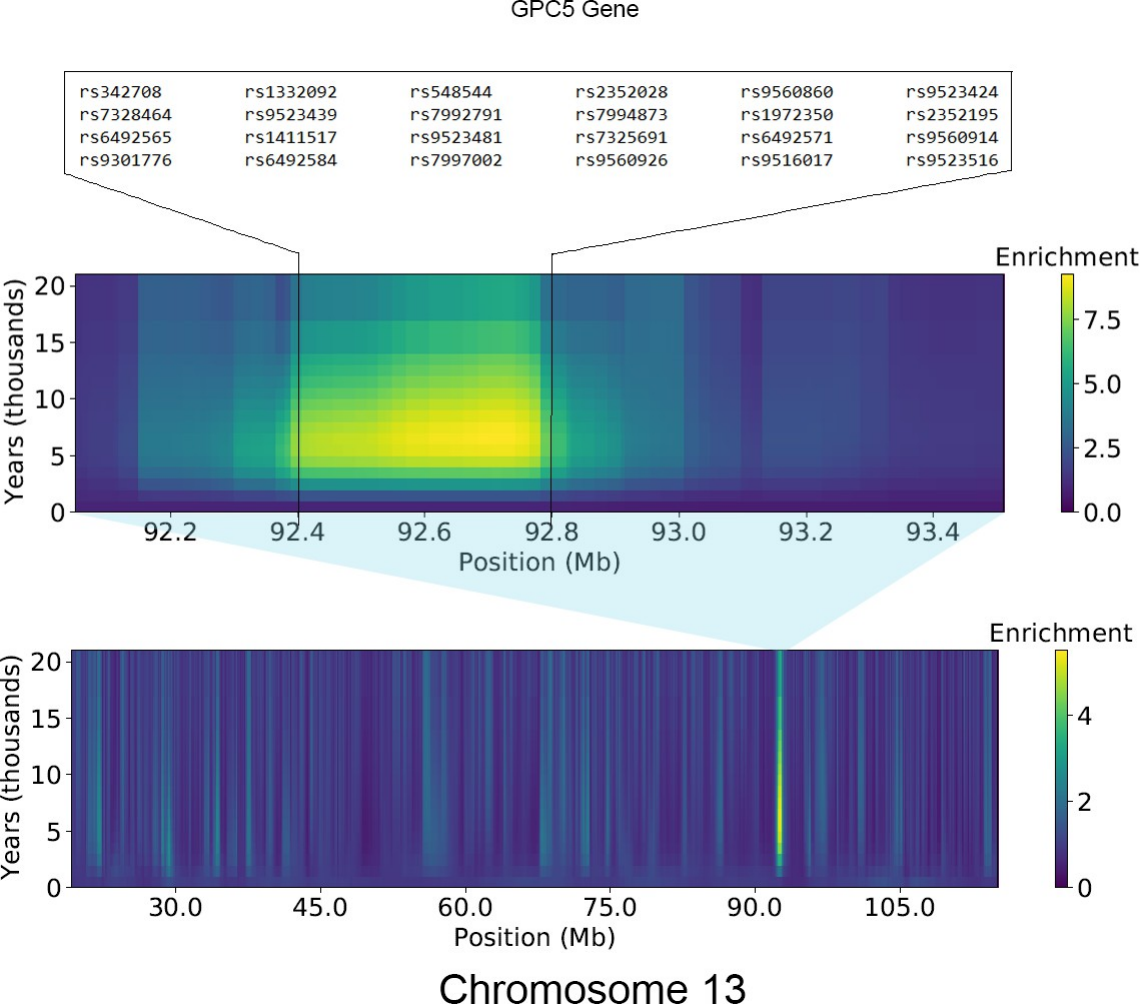
Ascertained Sequentially Markovian Coalescent (ASMC) results for chromosome 13. Showing with a greater resolution the region including the gene *GPC5* where the highest enrichment in recent coalescence events is concentrated on this chromosome.

In addition to the three regions described above, many other regions reported by Karlsson et al. (2013) were also identified in our study (Table 1). Most of these regions overlap with less than 4 genes in our analyses. As and example, Fig 3 shows the ASMC results for Chromosome 13, clearly showing a strong enrichment of recent coalescence events in a relatively narrow genomic interval including the *GPC5* gene. While there is very strong evidence pointing to the action of positive selection in these regions, an evaluation of the associations reported in the GWAS catalog (https://www.ebi.ac.uk/gwas/) indicates that most of these genes have pleiotropic effects and are associated with multiple traits in GWAS studies, so it is challenging to determine the specific selective factors driving these signals.

In contrast to the regions described above, which include a small number of genes, one of the regions identified in our study and also previously identified in South Asian [32] and East Asian samples [31] is an extremely gene-rich region spanning around 2.5 Mb located on Chromosome 16 between positions ~29.46 and 32.1 Mb (Tables 1 and S1 and S2 and S5 Fig). This region includes 78 genes and is characterized by the presence of gene regulatory factors (GRFs), including zinc-finger (ZNF) genes with a Krüppel-associated box (KRAB-ZNF). KRAB-ZNF genes have undergone extensive expansion in mammals and have been rapidly evolving in primates, and several of these genes are considered to be human-specific [31, 61]. Perdomo-Sabogal and Nowick (2019) [31] speculated that positive selection may have influenced diversity in several classes of GRF genes, thus playing an important role in local adaptation of human populations, and in their study, they identified numerous KRAB-ZNF clusters exhibiting evidence for positive selection in three human populations (the CEU, CHB and YRI 1KG samples). One of these GRF clusters overlaps with the chromosome 16 region identified in our study and it is possible that these regulatory genes have been the target of positive selection. However, it should be mentioned that this broad region also includes many non-GRF genes, thus making it difficult to pinpoint the target of selection. Interestingly, this region also includes the *VKORC1* gene, a very important pharmacogene that encodes a key enzyme in the vitamin K cycle and plays a key role in the coagulation pathway [62]. *VKORC1* is the pharmacological target of warfarin and previous studies have reported that positive selection may have played a role in the variability of anticoagulant response in humans [63, 64].

In addition to the signals shared between our study and Karlsson et al. (2013) [32] scan of positive selection in South Asians, we identified other outlier regions that have been reported in other studies (Table 1). These include a narrow region spanning a few kilobases on chromosome 22 (from 35.46 to 35.48 Mb) encompassing the homeobox *ISX* gene previously reported by Metspalu et al., (2011) [6], which has been reported to be a critical molecular mediator of the cross-talk between diet and immunity [65], and a region located on the short arm of chromosome 6 (from 29.5 to 33.1 Mb) previously reported by Liu et al. (2017) [7] and Suo et al. (2012) [29]. This corresponds to the Major Histocompatibility Complex (MHC) region, which is a well-known target of selection in the human genome [66, 67]. In our ASMC analysis, we observed the largest enrichment in the region around ~29.9Mb, which includes 7 genes (*SNORD32B*, *OR2H2*, *GABBR1*, *MOG*, *ZFP57*, *HLA-F*, *HLA-F-AS1*) (S9 Fig).

It is important to consider some of the limitations of this study. The first limitation is that our initial analysis was based on microarray-based data (approximately 300,000 markers) and not Whole Genome Sequencing (WGS) data, which is the ideal type of data to use for this type of studies. However, we compared the output of our analyses with the results obtained using the high-coverage genome sequencing data from two Indian samples of the 1KG Project (GIH and ITU), with highly consistent results: More than 90% of the regions identified in the microarray-based analysis are also outliers in the WGS analysis (S2 Table). The second limitation is that strategies based on the identification of outliers in the empirical distribution of the relevant parameters cannot fully guarantee that all these regions have been under the influence of positive selection. We tried to minimize the risks of false positives by selecting regions that are outliers in all the samples included in the study (WM-Tribes, WM-Castes, WM-All, 1KG-India) using more than one method. The microarray-based signals are very consistent with the WGS-based analysis and with our independent analysis using the ASMC method. It is also important to note that many of the regions identified in our study have been also reported in previous efforts to identify signatures of selection and from this perspective, the regions highlighted in Table 1 have particularly strong support. The third limitation is that, although we have identified regions that have been putatively under selection in South Asian populations, in many cases the regions include multiple genes, and it is not possible to identify which gene has been the target of selection. Similarly, even for the regions overlapping with only one gene, it is challenging to know what selective factors may have been involved as most of the genes are pleiotropic and have been associated with a broad range of traits. Despite these limitations, our study highlights numerous genomic regions that may have played a role in the adaptation of anatomically modern humans to novel environmental conditions after the out of Africa migration.

## Supporting information

S1 Fig The geographical location of the sample’s ancestries. This is a map with the geographical location of the sample’s ancestries that were analyzed in this study. GIH corresponds to Gujarati Indians in Houston, TX; and ITU corresponds to Indian Telugu in the UK.
(TIF)

S2 Fig Methods summary. Depicts a schematic representation of our approach. Additional details of the methods used in our analyses are provided in the main text.
(TIF)

S3 Fig Admixture graphs. Admixture graphs showing our two approaches. A) Admixture graph including a preIndia group resulting from admixture from a European and an Asian source. B) Admixture graph including preTribe and preCaste groups as a result of admixture between a European and Asian source.
(TIF)

S4 Fig ASMC results. ASMC, detailing in blue, regions found as putative signatures of natural selection in our study. The numbers in green indicate other studies where those regions were reported (1: Metspalu et al. 2011, 2: Suo et al. 2012, 3: Karlsson et al. 2013, 4: Liu et al. 2017, 5: Perdomo-Sabogal et al, 2019). In red we show genes present in regions with high enrichment but that were not found as an outlier based on the other six methods. A) chromosome 1, B) chromosome 2, C) chromosome 3, D) chromosome 4, E) chromosome 5, F) chromosome 6, G) chromosome 7, H) chromosome 8, I) chromosome 9, J) chromosome 10, K) chromosome 11, L) chromosome 12, M) chromosome 13, N) chromosome 14, O) chromosome 15, P) chromosome 16, Q) chromosome 17, R) chromosome 18, S) chromosome 19, T) chromosome 20, U) chromosome 21, V) chromosome 22.
(ZIP)

S5 Fig ASMC results for chromosome 16. ASMC results for chromosome 16, showing at the top, the genes identified in the region between 29.46Mb and 32.07Mb. In blue we list the genes within the top 0.5% signals and in red the genes within the top 0.1% signals identified for at least one method. The red squares highlight genes that have also been identified in other studies, as detailed in S1 Table.
(TIF)

S6 Fig Percentage of signals in the top 1% shared by pair of populations (WM Castes, WH Tribes, WH full sample, and India 1KG). The reference group is indicated in the Y-axis; for example, for the PBS method, 8.72% of the signals found in the WM Caste group are also found in the WM Tribe group, but just 7.78% of the signals identified in the WM Tribe group are found in the WM Caste group.
(TIF)

S7 Fig Percentage of signals in the top 1% shared by different methods. The reference method is indicated in the Y-axis; for example, 33.5% of the signals identified using PBS are also observed with xpEHH.
(TIF)

S8 Fig ASMC results for chromosome 2. ASMC results for chromosome 2, showing a zoom in the region with the biggest enrichment of recent coalescence events (96.2Mb to 98.4Mb), on the top we describe all the genes located within this region.
(TIF)

S9 Fig ASMC results for chromosome 6. ASMC results for chromosome 6, showing a zoom in the region from 29.6MbMb to 32.8Mb, on the top we describe the genes located within this region.
(TIF)

S1 Table Genomic regions identified in our genome-wide selection scan, including information on the genes overlapping each region. We also provide information about the results observed for the 1kgp high coverage data and overlap with other studies.
(XLSX)

S2 Table Summary details on the regions identified in our genome-wide selection scan. We also provide information about the results observed for the 1kgp high coverage data and signals reported in the PopHuman Browser for the iHS statistic, as well as overlap with other studies.
(XLSX)
