# Supplementary figures and images for: Identifying signatures of natural selection in Indian populations
Source: PLoS One. 2022 Aug 4;17(8):e0271767. doi: 10.1371/journal.pone.0271767 (PMC9352006; doi:10.1371/journal.pone.0271767)

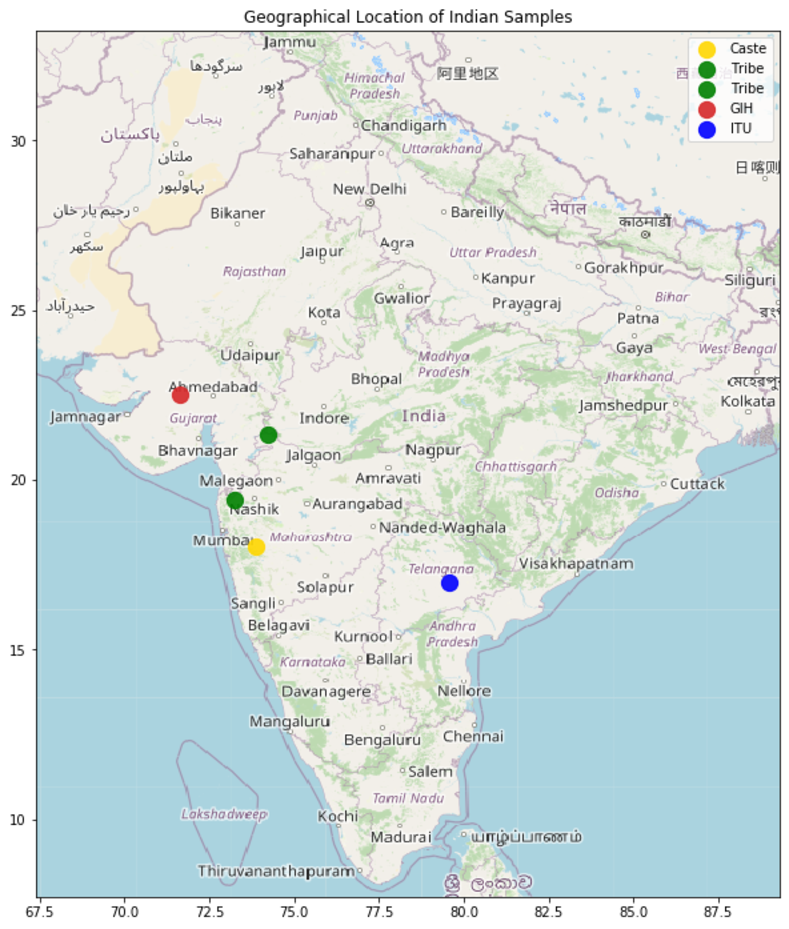

Supplement: S1 Fig — This is a map with the geographical location of the sample’s ancestries that were analyzed in this study. GIH corresponds to Gujarati Indians in Houston, TX; and ITU corresponds to Indian Telugu in the UK. (TIF) [file pone.0271767.s001.tif]

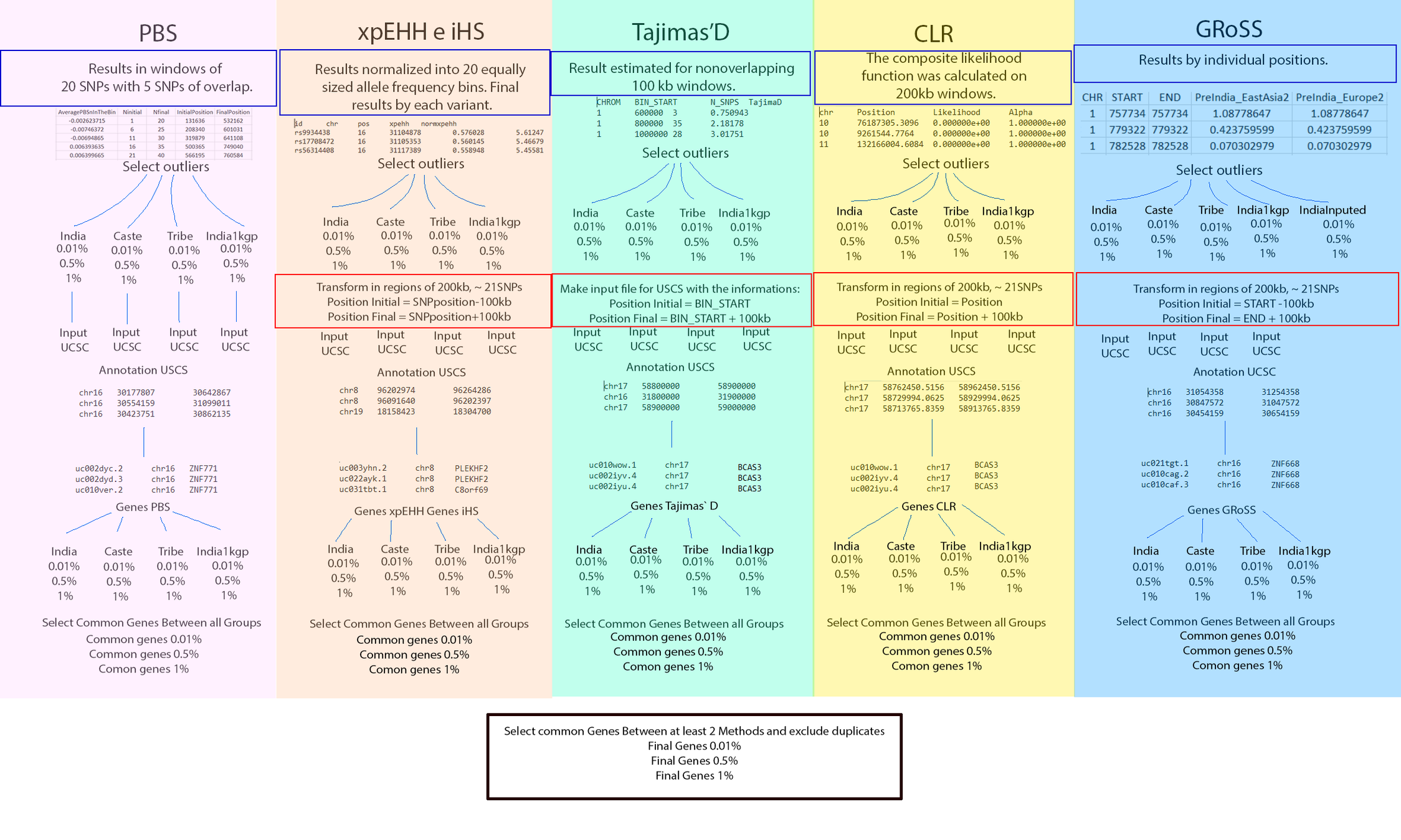

Supplement: S2 Fig — Depicts a schematic representation of our approach. Additional details of the methods used in our analyses are provided in the main text. (TIF) [file pone.0271767.s002.tif]

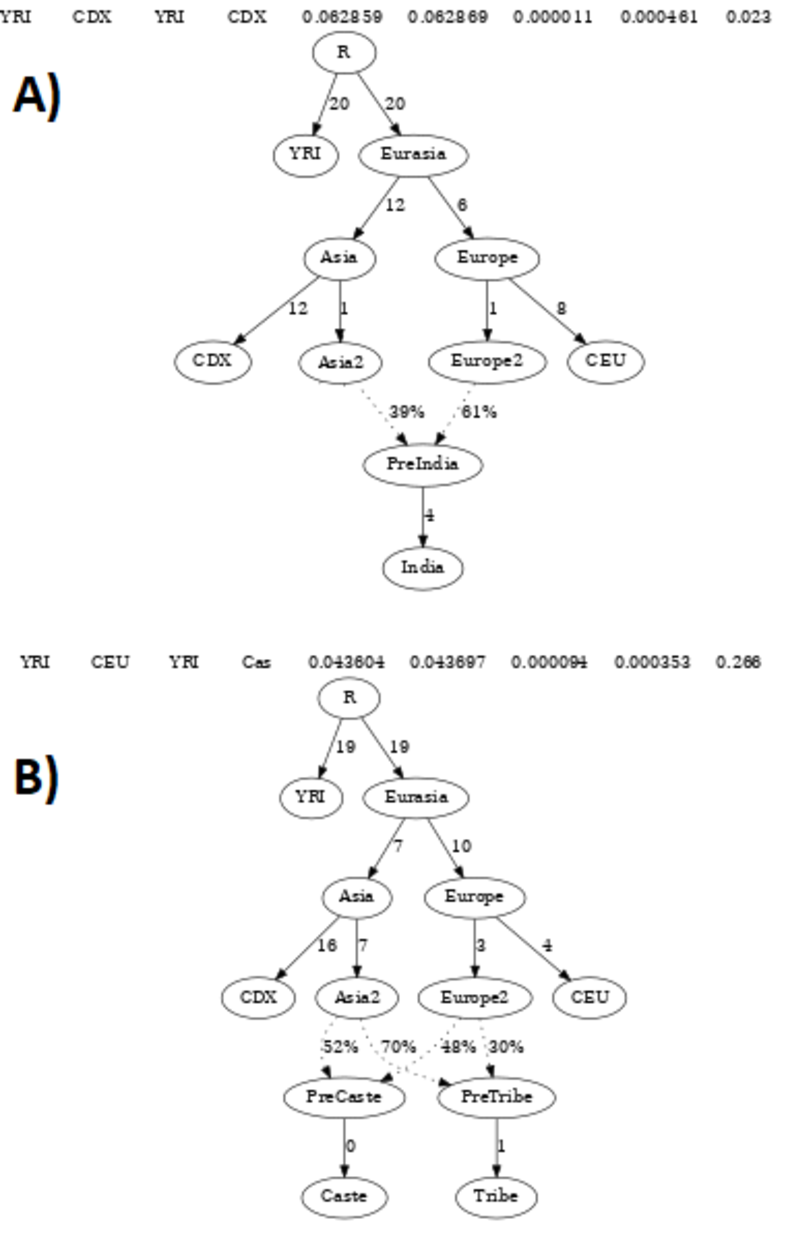

Supplement: S3 Fig — Admixture graphs showing our two approaches. A) Admixture graph including a preIndia group resulting from admixture from a European and an Asian source. B) Admixture graph including preTribe and preCaste groups as a result of admixture between a European and Asian source. (TIF) [file pone.0271767.s003.tif]

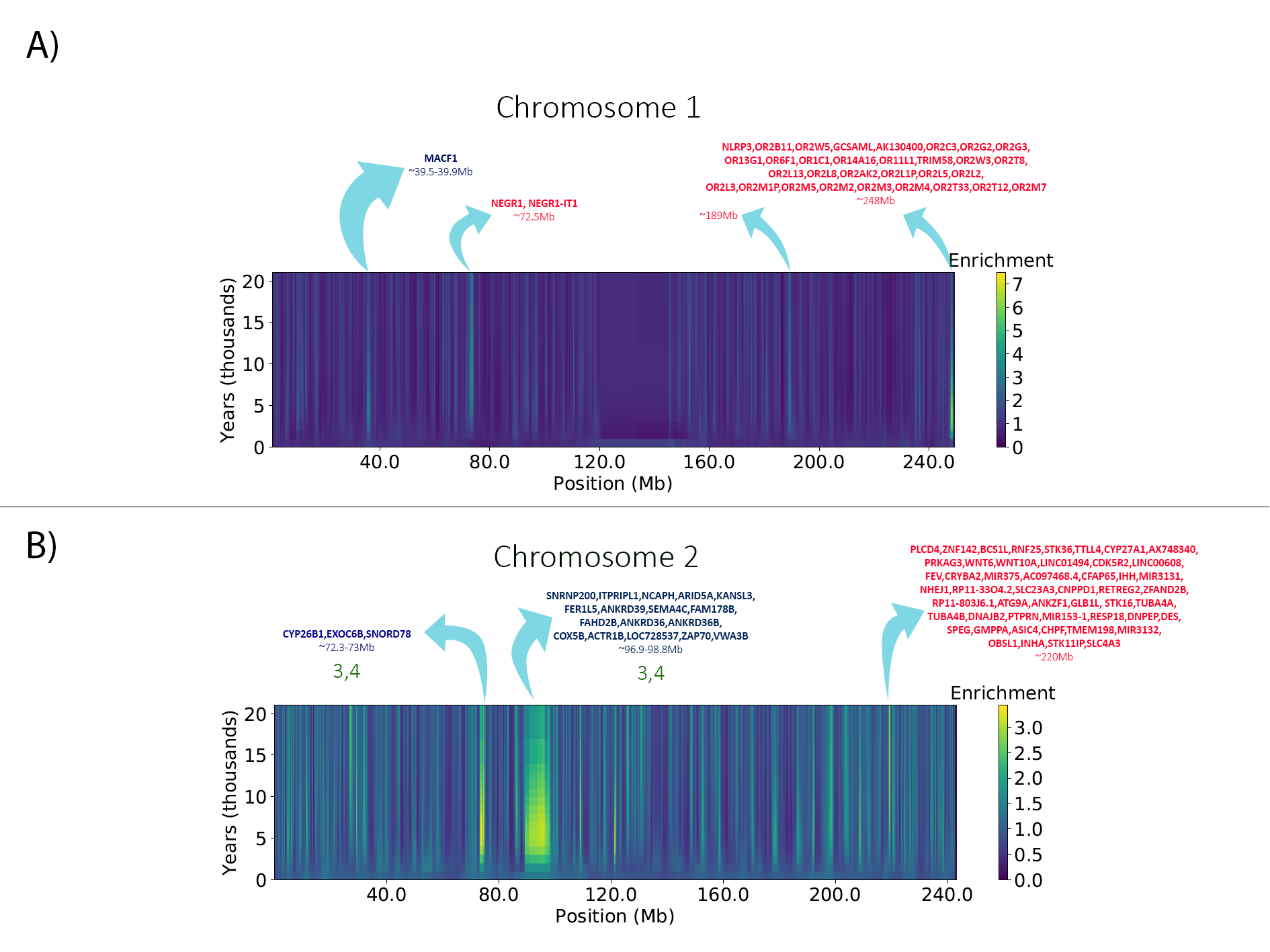

Supplement: S4 Fig — ASMC, detailing in blue, regions found as putative signatures of natural selection in our study. The numbers in green indicate other studies where those regions were reported (1: Metspalu et al. 2011, 2: Suo et al. 2012, 3: Karlsson et al. 2013, 4: Liu et al. 2017, 5: Perdomo-Sabogal et al, 2019). In red we show genes present in regions with high enrichment but that were not found as an outlier based on the other six methods. A) chromosome 1, B) chromosome 2, C) chromosome 3, D) chromosome 4, E) chromosome 5, F) chromosome 6, G) chromosome 7, H) chromosome 8, I) chromosome 9, J) chromosome 10, K) chromosome 11, L) chromosome 12, M) chromosome 13, N) chromosome 14, O) chromosome 15, P) chromosome 16, Q) chromosome 17, R) chromosome 18, S) chromosome 19, T) chromosome 20, U) chromosome 21, V) chromosome 22. (ZIP) [file pone.0271767.s004.zip › S4 Figure A-B.tif]

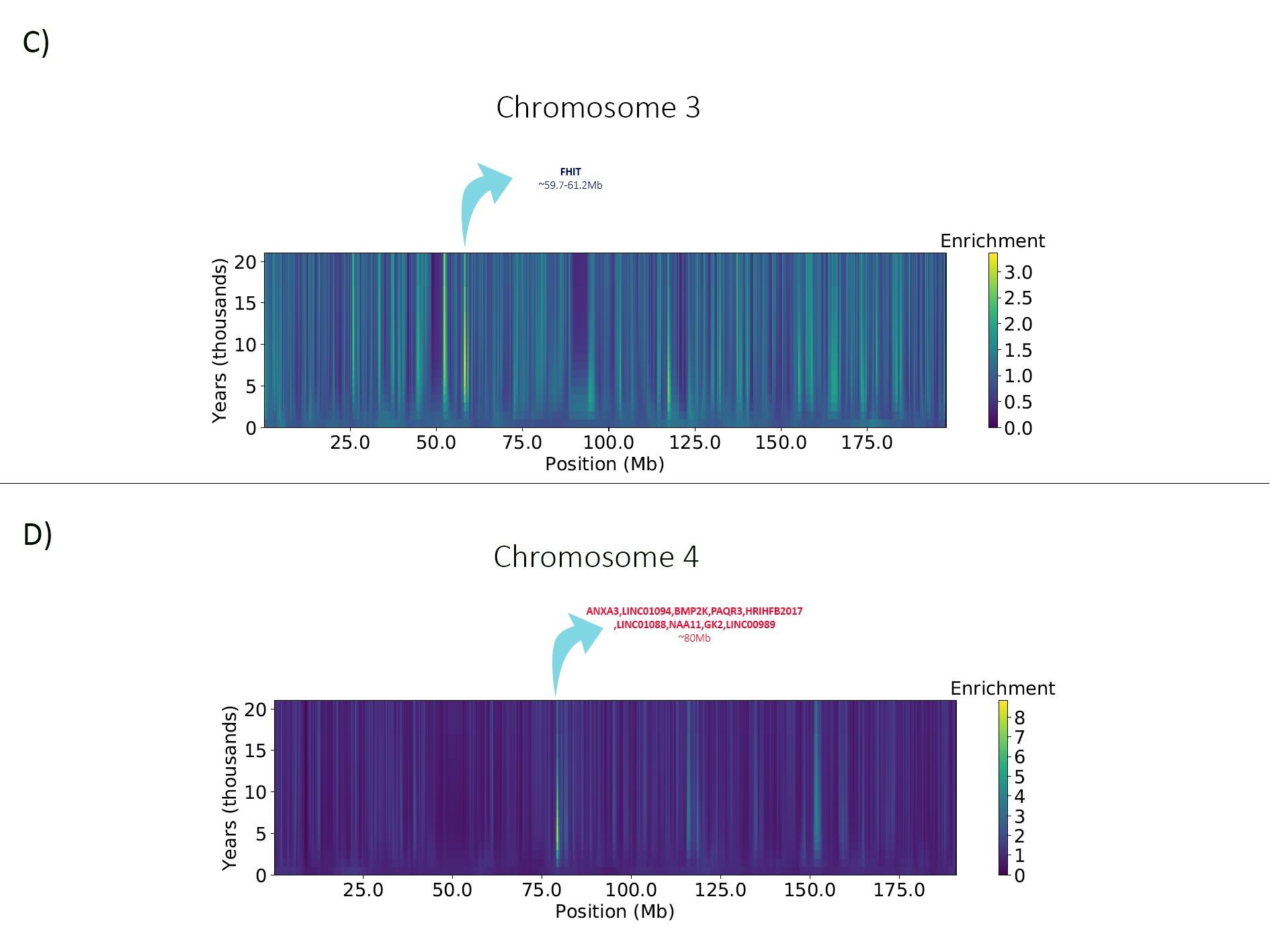

Supplement: S4 Fig — ASMC, detailing in blue, regions found as putative signatures of natural selection in our study. The numbers in green indicate other studies where those regions were reported (1: Metspalu et al. 2011, 2: Suo et al. 2012, 3: Karlsson et al. 2013, 4: Liu et al. 2017, 5: Perdomo-Sabogal et al, 2019). In red we show genes present in regions with high enrichment but that were not found as an outlier based on the other six methods. A) chromosome 1, B) chromosome 2, C) chromosome 3, D) chromosome 4, E) chromosome 5, F) chromosome 6, G) chromosome 7, H) chromosome 8, I) chromosome 9, J) chromosome 10, K) chromosome 11, L) chromosome 12, M) chromosome 13, N) chromosome 14, O) chromosome 15, P) chromosome 16, Q) chromosome 17, R) chromosome 18, S) chromosome 19, T) chromosome 20, U) chromosome 21, V) chromosome 22. (ZIP) [file pone.0271767.s004.zip › S4 Figure C-D.tif]

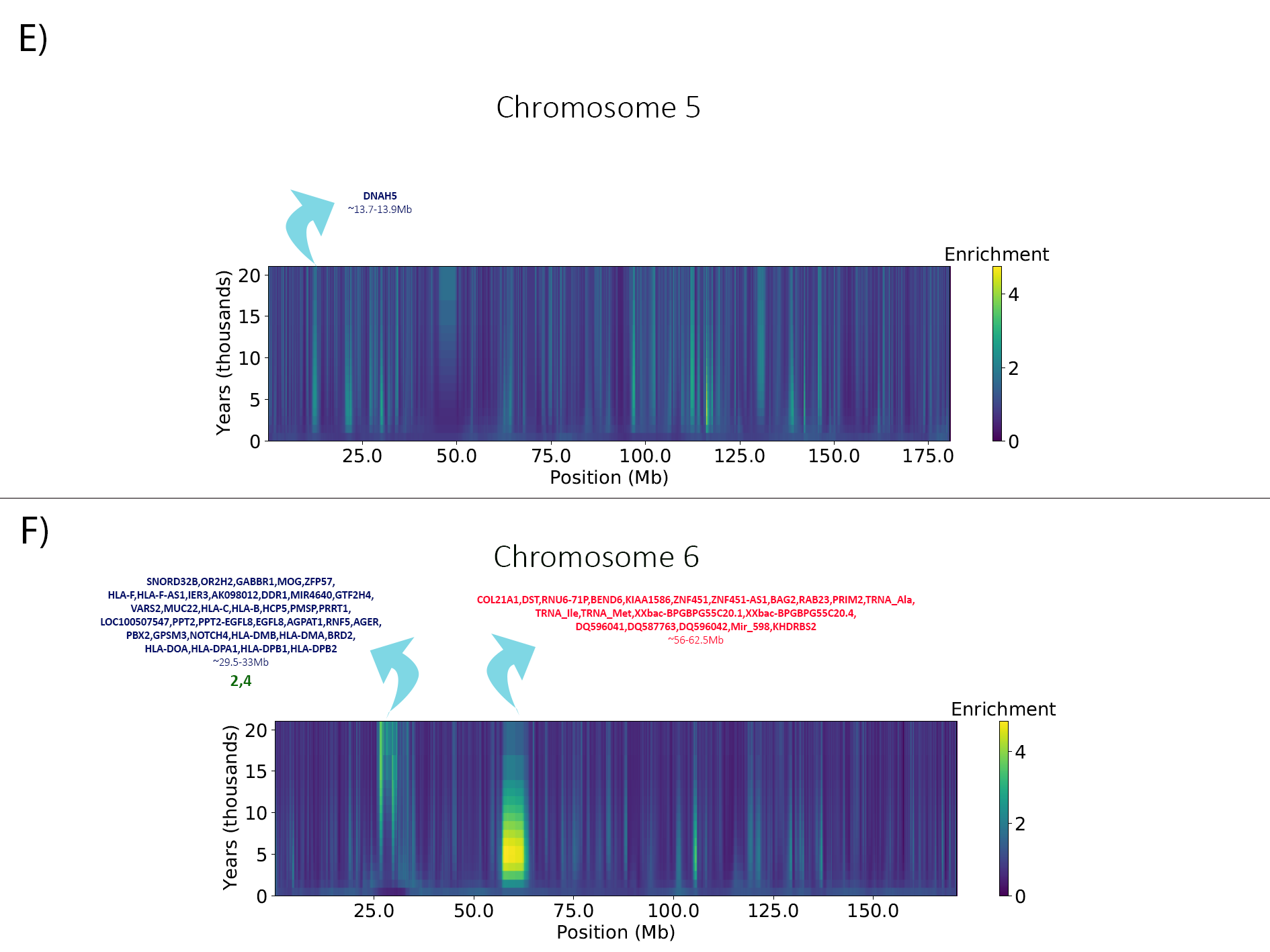

Supplement: S4 Fig — ASMC, detailing in blue, regions found as putative signatures of natural selection in our study. The numbers in green indicate other studies where those regions were reported (1: Metspalu et al. 2011, 2: Suo et al. 2012, 3: Karlsson et al. 2013, 4: Liu et al. 2017, 5: Perdomo-Sabogal et al, 2019). In red we show genes present in regions with high enrichment but that were not found as an outlier based on the other six methods. A) chromosome 1, B) chromosome 2, C) chromosome 3, D) chromosome 4, E) chromosome 5, F) chromosome 6, G) chromosome 7, H) chromosome 8, I) chromosome 9, J) chromosome 10, K) chromosome 11, L) chromosome 12, M) chromosome 13, N) chromosome 14, O) chromosome 15, P) chromosome 16, Q) chromosome 17, R) chromosome 18, S) chromosome 19, T) chromosome 20, U) chromosome 21, V) chromosome 22. (ZIP) [file pone.0271767.s004.zip › S4 Figure E-F.tif]

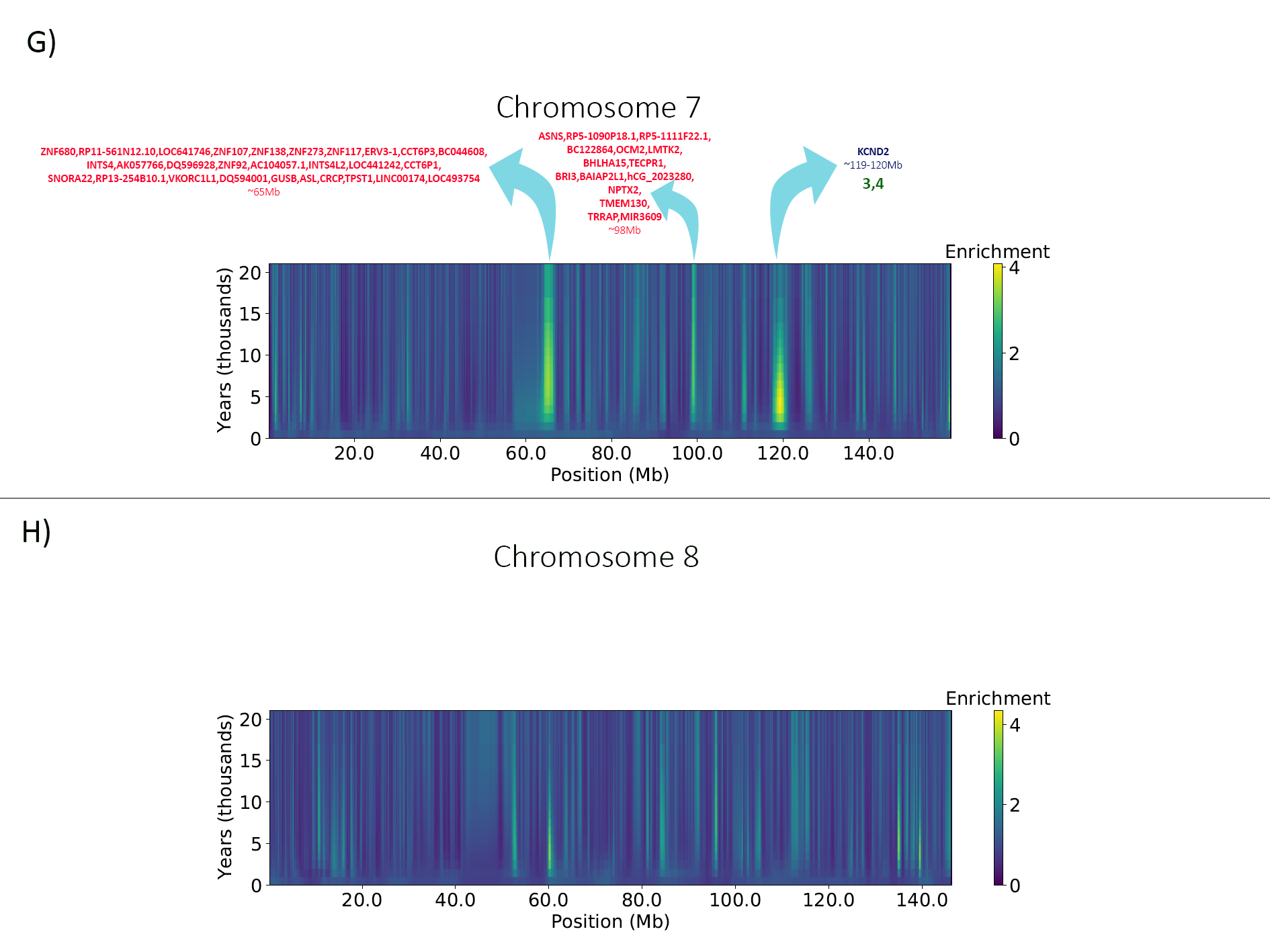

Supplement: S4 Fig — ASMC, detailing in blue, regions found as putative signatures of natural selection in our study. The numbers in green indicate other studies where those regions were reported (1: Metspalu et al. 2011, 2: Suo et al. 2012, 3: Karlsson et al. 2013, 4: Liu et al. 2017, 5: Perdomo-Sabogal et al, 2019). In red we show genes present in regions with high enrichment but that were not found as an outlier based on the other six methods. A) chromosome 1, B) chromosome 2, C) chromosome 3, D) chromosome 4, E) chromosome 5, F) chromosome 6, G) chromosome 7, H) chromosome 8, I) chromosome 9, J) chromosome 10, K) chromosome 11, L) chromosome 12, M) chromosome 13, N) chromosome 14, O) chromosome 15, P) chromosome 16, Q) chromosome 17, R) chromosome 18, S) chromosome 19, T) chromosome 20, U) chromosome 21, V) chromosome 22. (ZIP) [file pone.0271767.s004.zip › S4 Figure G-H.tif]

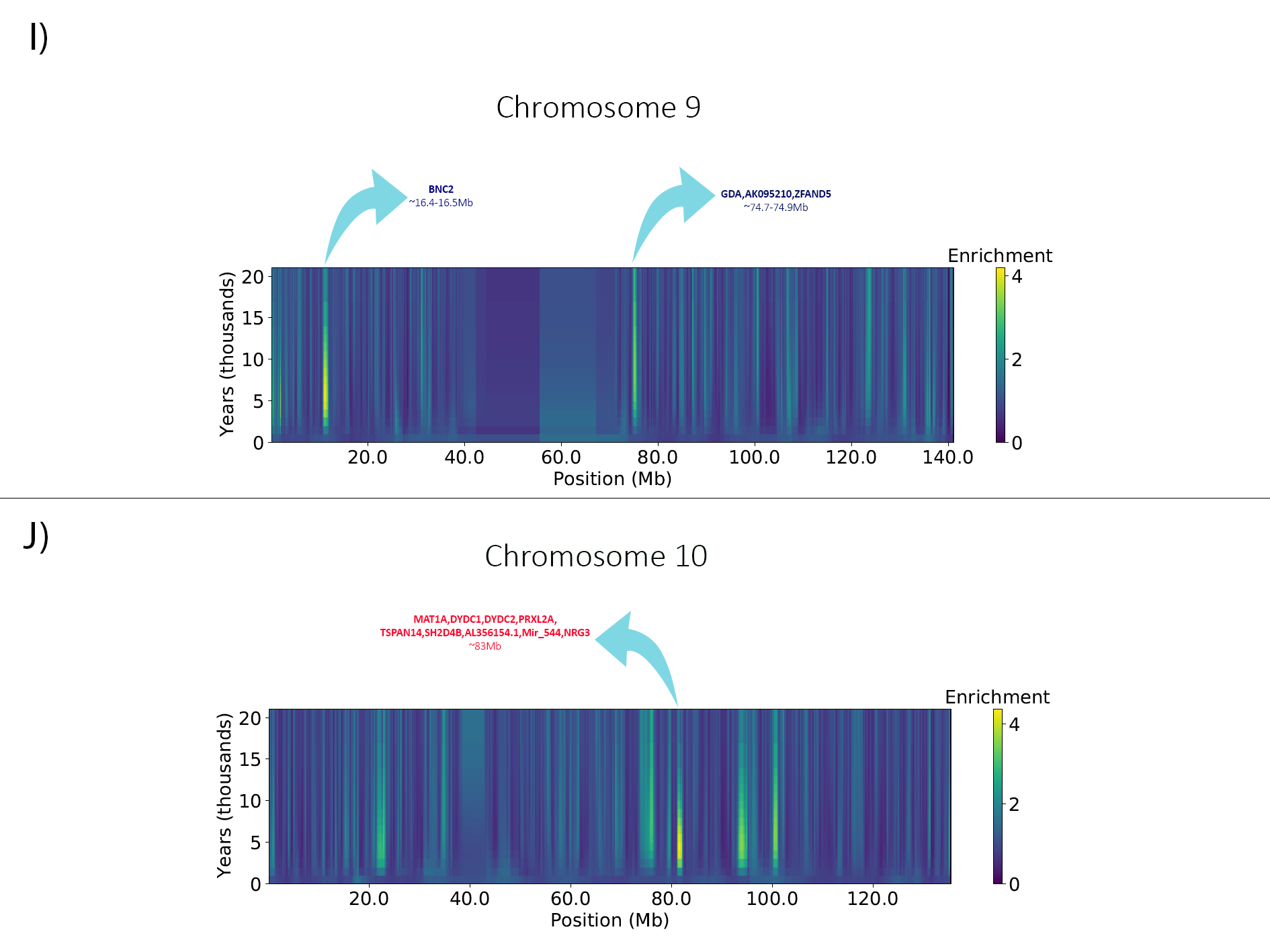

Supplement: S4 Fig — ASMC, detailing in blue, regions found as putative signatures of natural selection in our study. The numbers in green indicate other studies where those regions were reported (1: Metspalu et al. 2011, 2: Suo et al. 2012, 3: Karlsson et al. 2013, 4: Liu et al. 2017, 5: Perdomo-Sabogal et al, 2019). In red we show genes present in regions with high enrichment but that were not found as an outlier based on the other six methods. A) chromosome 1, B) chromosome 2, C) chromosome 3, D) chromosome 4, E) chromosome 5, F) chromosome 6, G) chromosome 7, H) chromosome 8, I) chromosome 9, J) chromosome 10, K) chromosome 11, L) chromosome 12, M) chromosome 13, N) chromosome 14, O) chromosome 15, P) chromosome 16, Q) chromosome 17, R) chromosome 18, S) chromosome 19, T) chromosome 20, U) chromosome 21, V) chromosome 22. (ZIP) [file pone.0271767.s004.zip › S4 Figure I-J.tif]

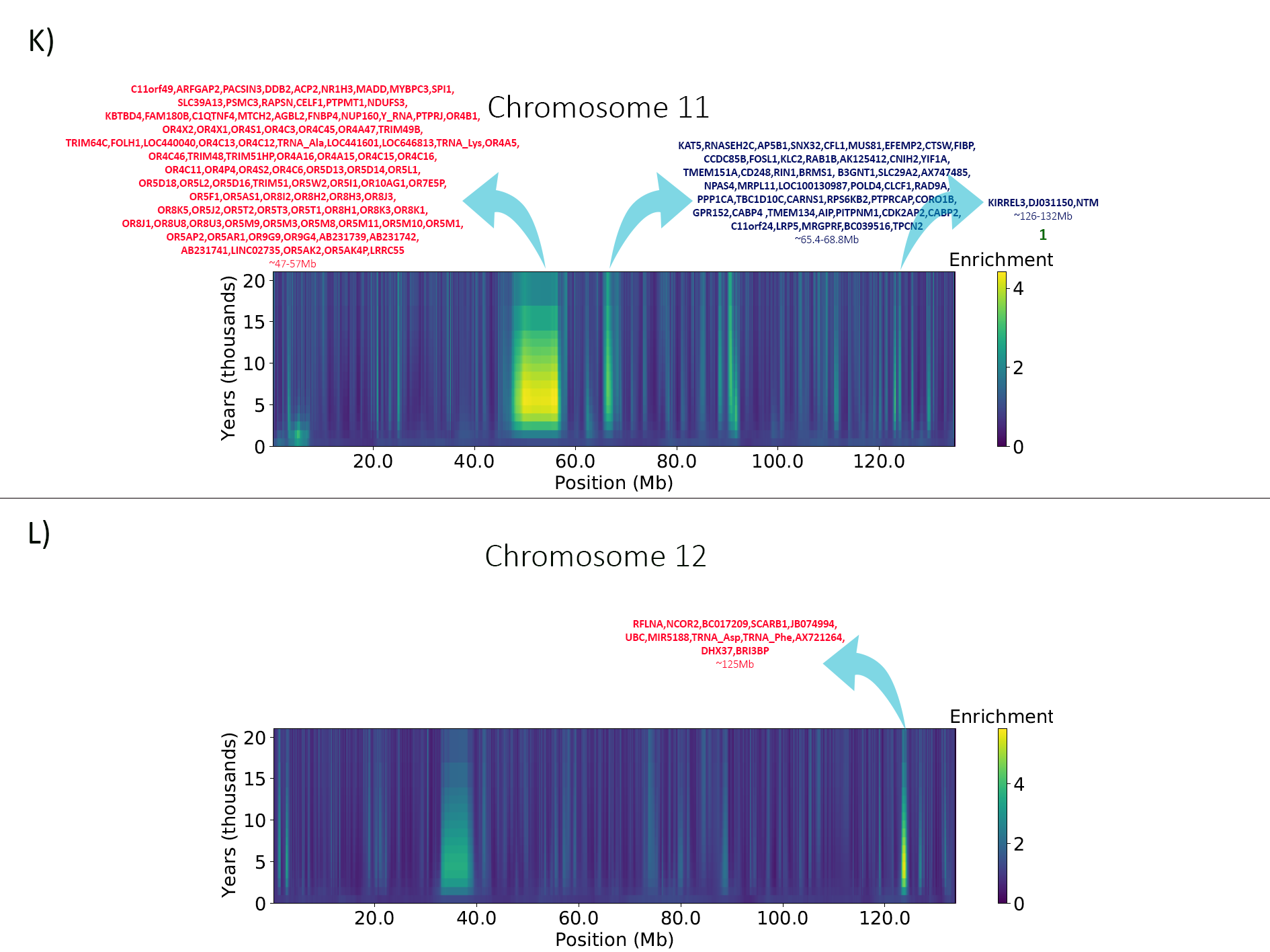

Supplement: S4 Fig — ASMC, detailing in blue, regions found as putative signatures of natural selection in our study. The numbers in green indicate other studies where those regions were reported (1: Metspalu et al. 2011, 2: Suo et al. 2012, 3: Karlsson et al. 2013, 4: Liu et al. 2017, 5: Perdomo-Sabogal et al, 2019). In red we show genes present in regions with high enrichment but that were not found as an outlier based on the other six methods. A) chromosome 1, B) chromosome 2, C) chromosome 3, D) chromosome 4, E) chromosome 5, F) chromosome 6, G) chromosome 7, H) chromosome 8, I) chromosome 9, J) chromosome 10, K) chromosome 11, L) chromosome 12, M) chromosome 13, N) chromosome 14, O) chromosome 15, P) chromosome 16, Q) chromosome 17, R) chromosome 18, S) chromosome 19, T) chromosome 20, U) chromosome 21, V) chromosome 22. (ZIP) [file pone.0271767.s004.zip › S4 Figure K-L.tif]

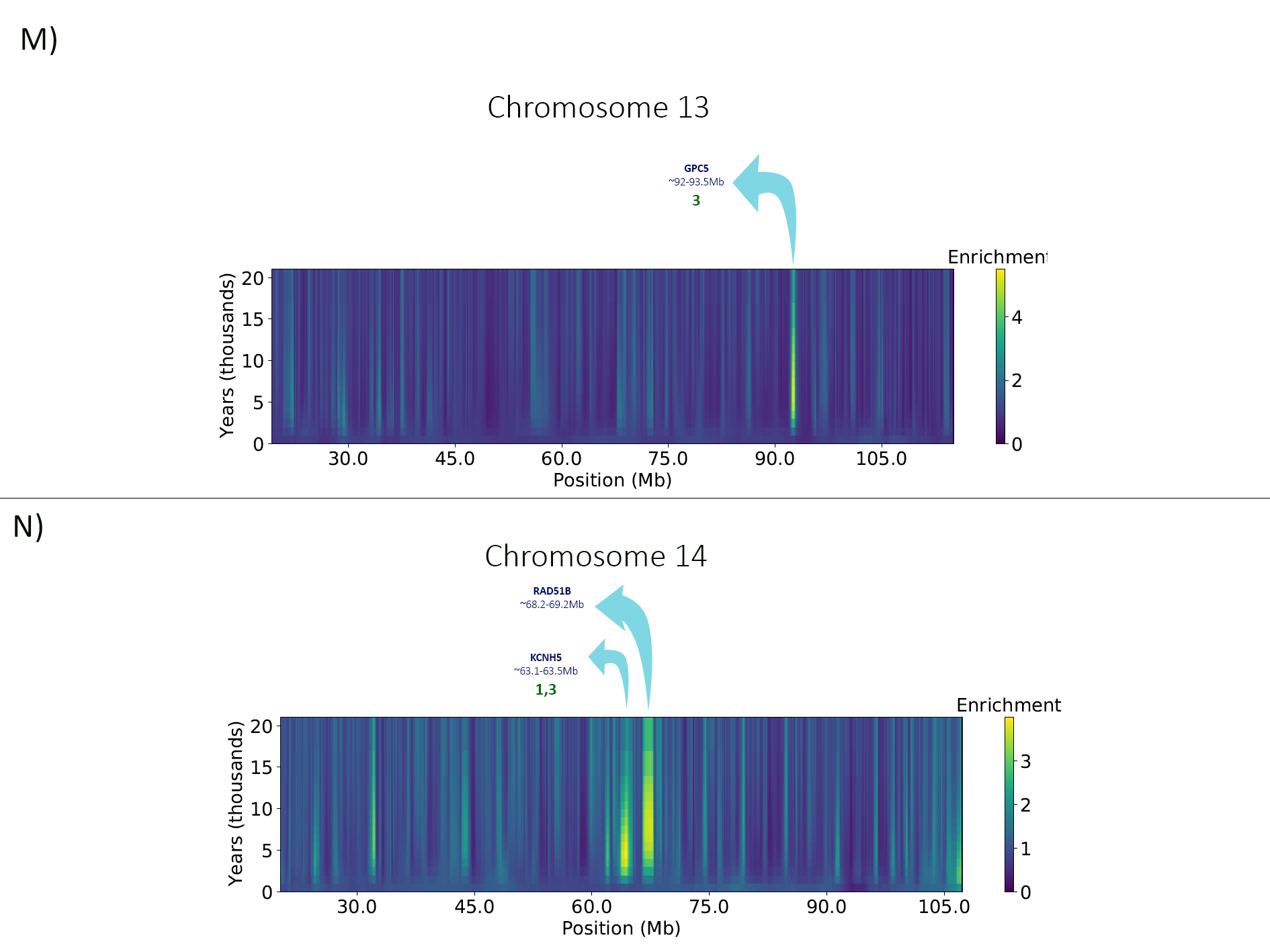

Supplement: S4 Fig — ASMC, detailing in blue, regions found as putative signatures of natural selection in our study. The numbers in green indicate other studies where those regions were reported (1: Metspalu et al. 2011, 2: Suo et al. 2012, 3: Karlsson et al. 2013, 4: Liu et al. 2017, 5: Perdomo-Sabogal et al, 2019). In red we show genes present in regions with high enrichment but that were not found as an outlier based on the other six methods. A) chromosome 1, B) chromosome 2, C) chromosome 3, D) chromosome 4, E) chromosome 5, F) chromosome 6, G) chromosome 7, H) chromosome 8, I) chromosome 9, J) chromosome 10, K) chromosome 11, L) chromosome 12, M) chromosome 13, N) chromosome 14, O) chromosome 15, P) chromosome 16, Q) chromosome 17, R) chromosome 18, S) chromosome 19, T) chromosome 20, U) chromosome 21, V) chromosome 22. (ZIP) [file pone.0271767.s004.zip › S4 Figure M-N.tif]

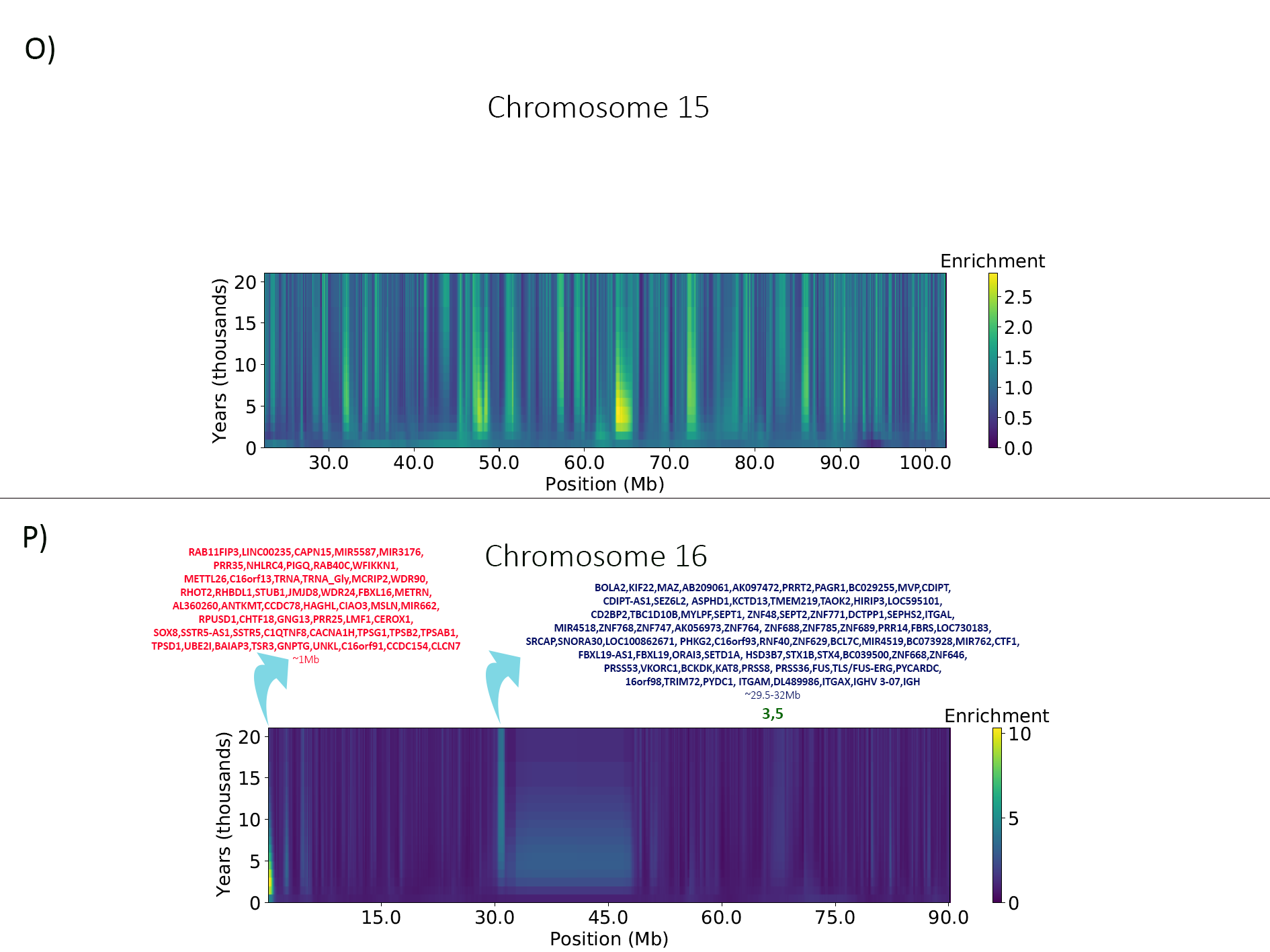

Supplement: S4 Fig — ASMC, detailing in blue, regions found as putative signatures of natural selection in our study. The numbers in green indicate other studies where those regions were reported (1: Metspalu et al. 2011, 2: Suo et al. 2012, 3: Karlsson et al. 2013, 4: Liu et al. 2017, 5: Perdomo-Sabogal et al, 2019). In red we show genes present in regions with high enrichment but that were not found as an outlier based on the other six methods. A) chromosome 1, B) chromosome 2, C) chromosome 3, D) chromosome 4, E) chromosome 5, F) chromosome 6, G) chromosome 7, H) chromosome 8, I) chromosome 9, J) chromosome 10, K) chromosome 11, L) chromosome 12, M) chromosome 13, N) chromosome 14, O) chromosome 15, P) chromosome 16, Q) chromosome 17, R) chromosome 18, S) chromosome 19, T) chromosome 20, U) chromosome 21, V) chromosome 22. (ZIP) [file pone.0271767.s004.zip › S4 Figure O-P.tif]

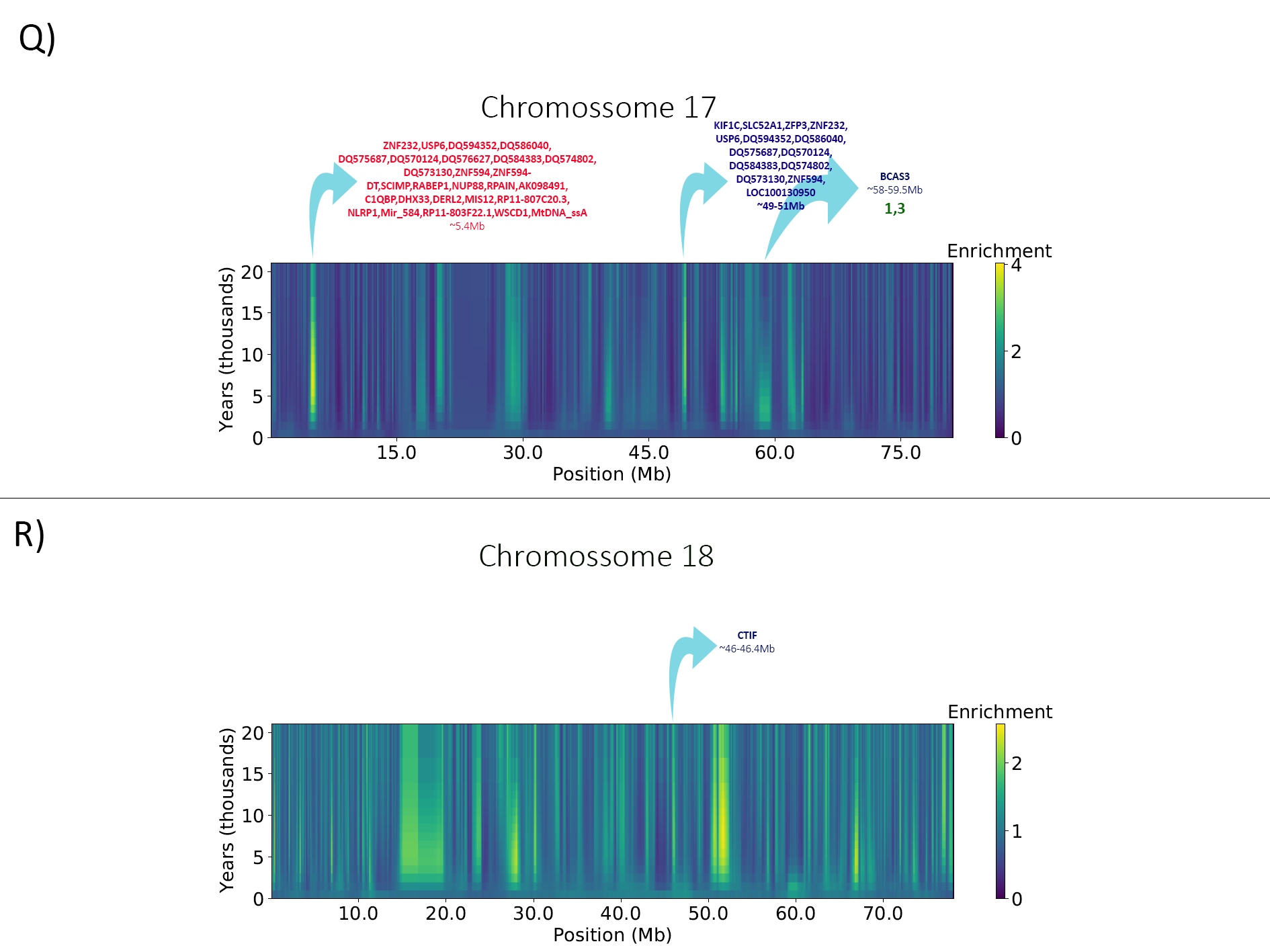

Supplement: S4 Fig — ASMC, detailing in blue, regions found as putative signatures of natural selection in our study. The numbers in green indicate other studies where those regions were reported (1: Metspalu et al. 2011, 2: Suo et al. 2012, 3: Karlsson et al. 2013, 4: Liu et al. 2017, 5: Perdomo-Sabogal et al, 2019). In red we show genes present in regions with high enrichment but that were not found as an outlier based on the other six methods. A) chromosome 1, B) chromosome 2, C) chromosome 3, D) chromosome 4, E) chromosome 5, F) chromosome 6, G) chromosome 7, H) chromosome 8, I) chromosome 9, J) chromosome 10, K) chromosome 11, L) chromosome 12, M) chromosome 13, N) chromosome 14, O) chromosome 15, P) chromosome 16, Q) chromosome 17, R) chromosome 18, S) chromosome 19, T) chromosome 20, U) chromosome 21, V) chromosome 22. (ZIP) [file pone.0271767.s004.zip › S4 Figure Q-R.tif]

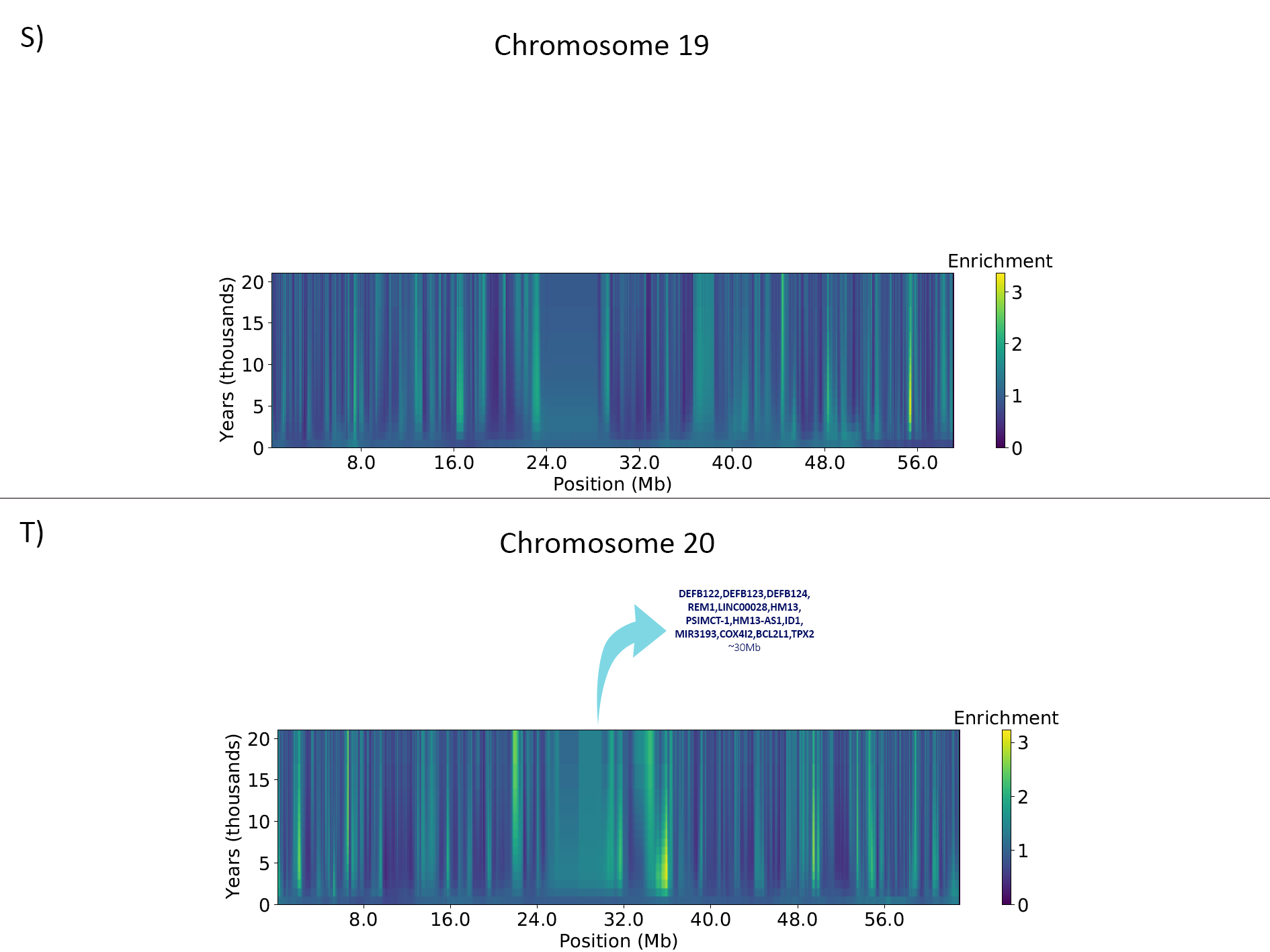

Supplement: S4 Fig — ASMC, detailing in blue, regions found as putative signatures of natural selection in our study. The numbers in green indicate other studies where those regions were reported (1: Metspalu et al. 2011, 2: Suo et al. 2012, 3: Karlsson et al. 2013, 4: Liu et al. 2017, 5: Perdomo-Sabogal et al, 2019). In red we show genes present in regions with high enrichment but that were not found as an outlier based on the other six methods. A) chromosome 1, B) chromosome 2, C) chromosome 3, D) chromosome 4, E) chromosome 5, F) chromosome 6, G) chromosome 7, H) chromosome 8, I) chromosome 9, J) chromosome 10, K) chromosome 11, L) chromosome 12, M) chromosome 13, N) chromosome 14, O) chromosome 15, P) chromosome 16, Q) chromosome 17, R) chromosome 18, S) chromosome 19, T) chromosome 20, U) chromosome 21, V) chromosome 22. (ZIP) [file pone.0271767.s004.zip › S4 Figure S-T.tif]

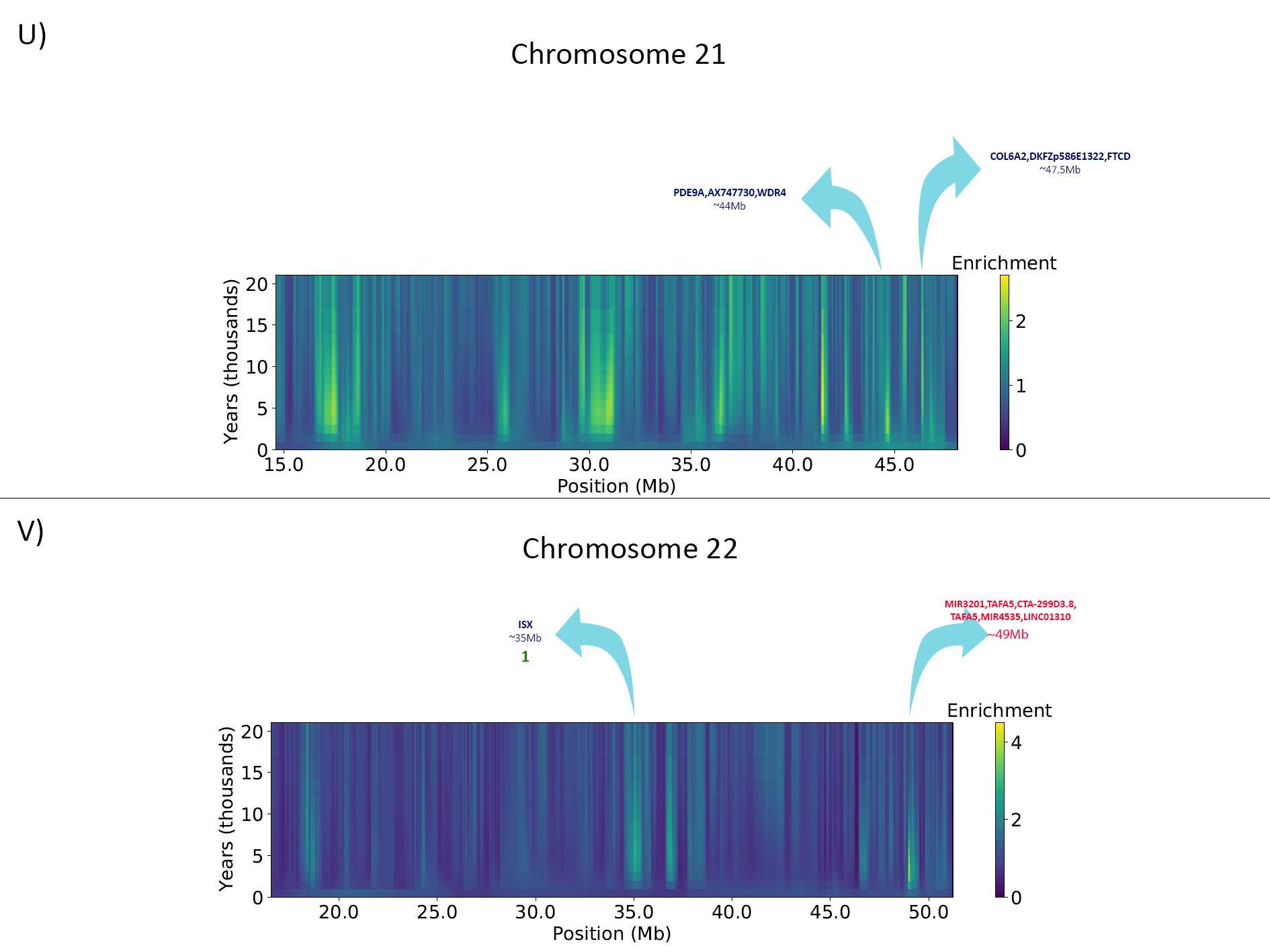

Supplement: S4 Fig — ASMC, detailing in blue, regions found as putative signatures of natural selection in our study. The numbers in green indicate other studies where those regions were reported (1: Metspalu et al. 2011, 2: Suo et al. 2012, 3: Karlsson et al. 2013, 4: Liu et al. 2017, 5: Perdomo-Sabogal et al, 2019). In red we show genes present in regions with high enrichment but that were not found as an outlier based on the other six methods. A) chromosome 1, B) chromosome 2, C) chromosome 3, D) chromosome 4, E) chromosome 5, F) chromosome 6, G) chromosome 7, H) chromosome 8, I) chromosome 9, J) chromosome 10, K) chromosome 11, L) chromosome 12, M) chromosome 13, N) chromosome 14, O) chromosome 15, P) chromosome 16, Q) chromosome 17, R) chromosome 18, S) chromosome 19, T) chromosome 20, U) chromosome 21, V) chromosome 22. (ZIP) [file pone.0271767.s004.zip › S4 Figure U-V.tif]

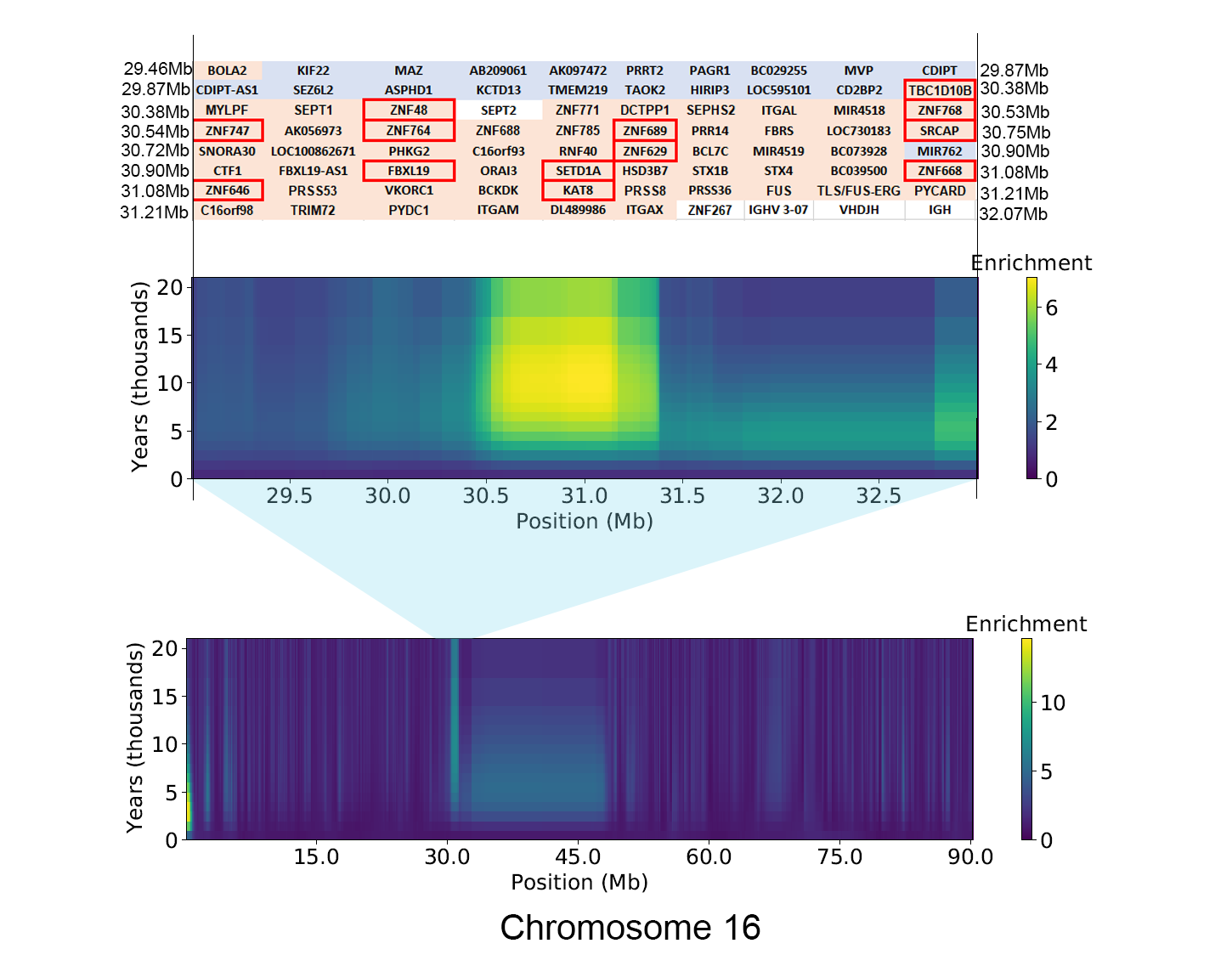

Supplement: S5 Fig — ASMC results for chromosome 16, showing at the top, the genes identified in the region between 29.46Mb and 32.07Mb. In blue we list the genes within the top 0.5% signals and in red the genes within the top 0.1% signals identified for at least one method. The red squares highlight genes that have also been identified in other studies, as detailed in S1 Table. (TIF) [file pone.0271767.s005.tif]

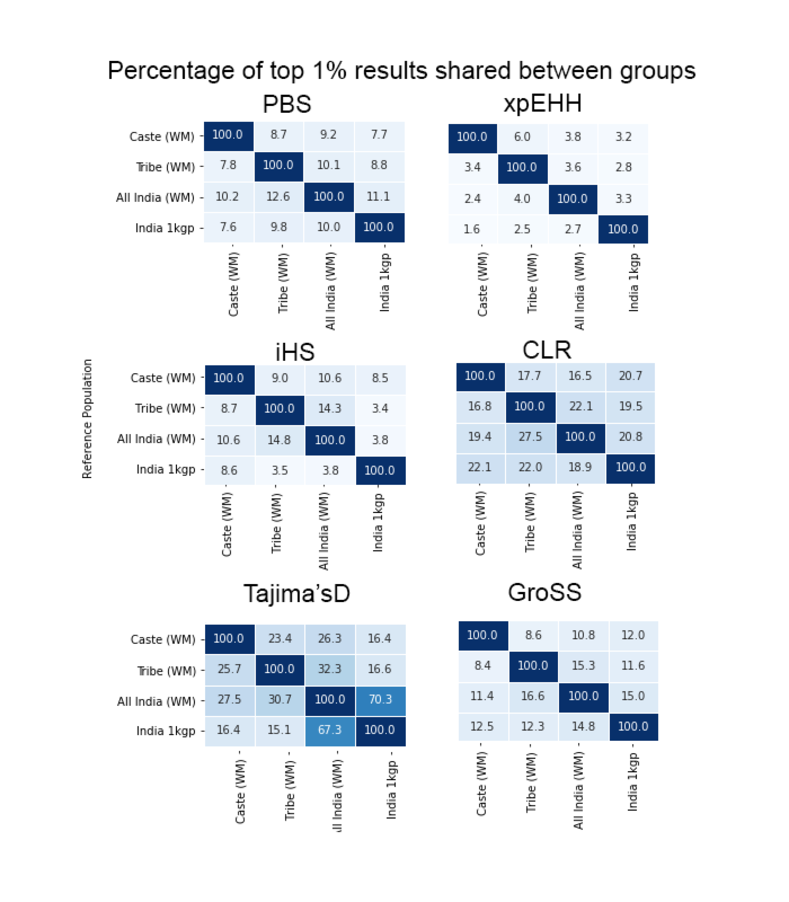

Supplement: S6 Fig — The reference group is indicated in the Y-axis; for example, for the PBS method, 8.72% of the signals found in the WM Caste group are also found in the WM Tribe group, but just 7.78% of the signals identified in the WM Tribe group are found in the WM Caste group. (TIF) [file pone.0271767.s006.tif]

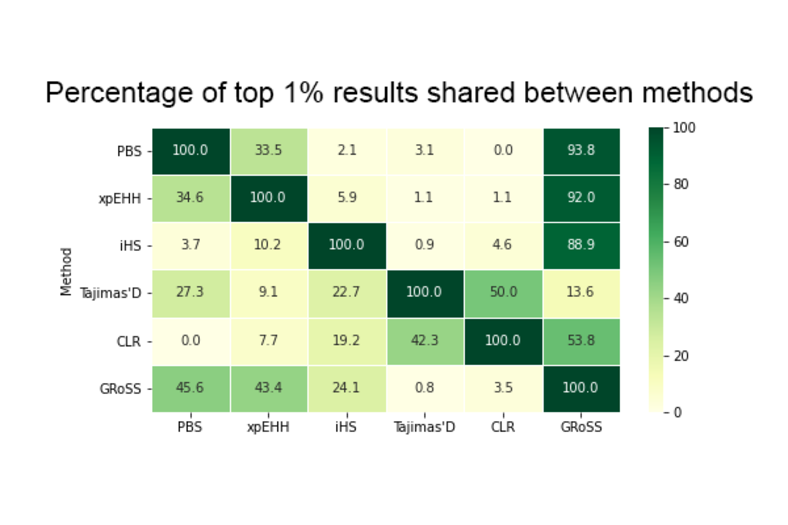

Supplement: S7 Fig — The reference method is indicated in the Y-axis; for example, 33.5% of the signals identified using PBS are also observed with xpEHH. (TIF) [file pone.0271767.s007.tif]

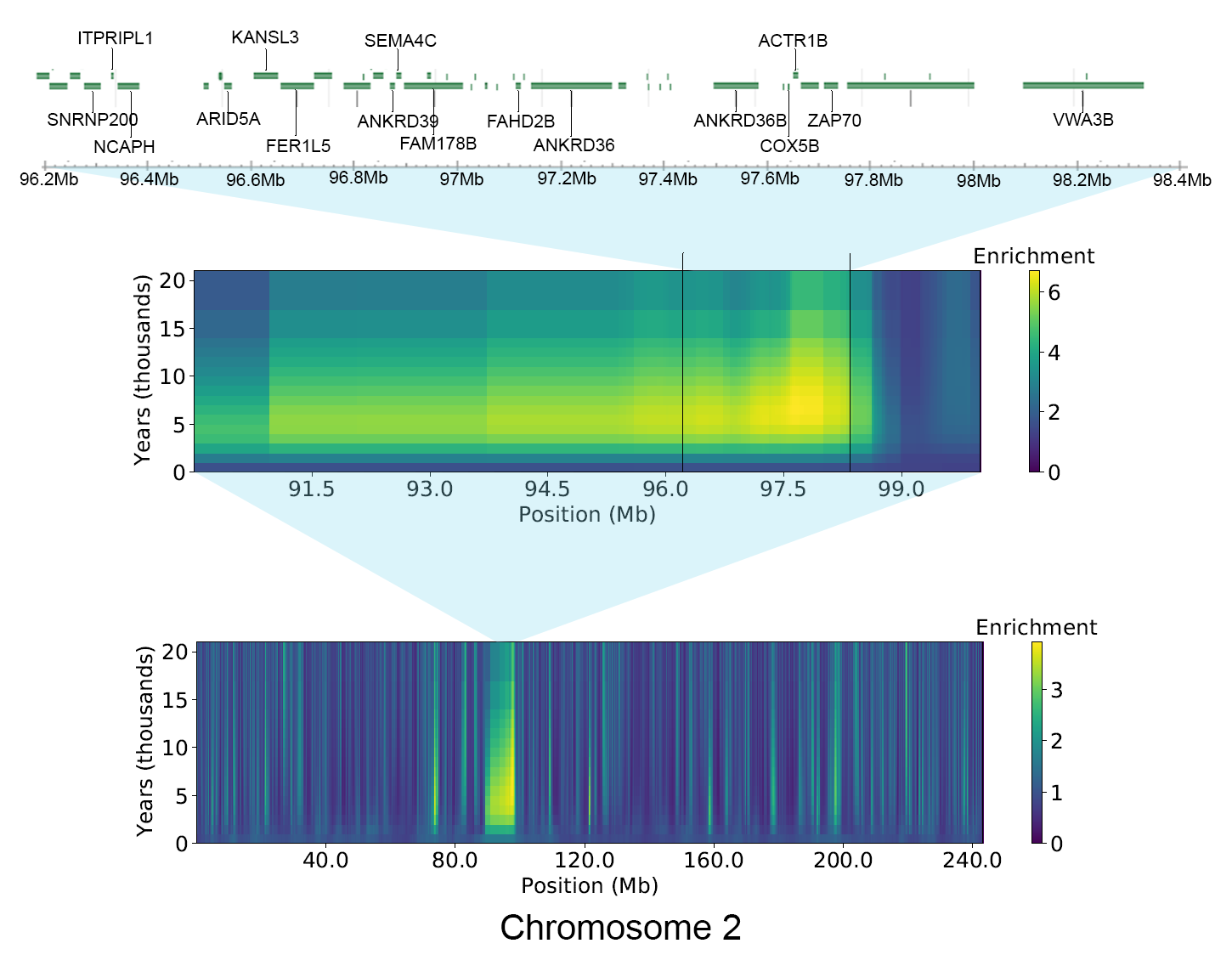

Supplement: S8 Fig — ASMC results for chromosome 2, showing a zoom in the region with the biggest enrichment of recent coalescence events (96.2Mb to 98.4Mb), on the top we describe all the genes located within this region. (TIF) [file pone.0271767.s008.tif]

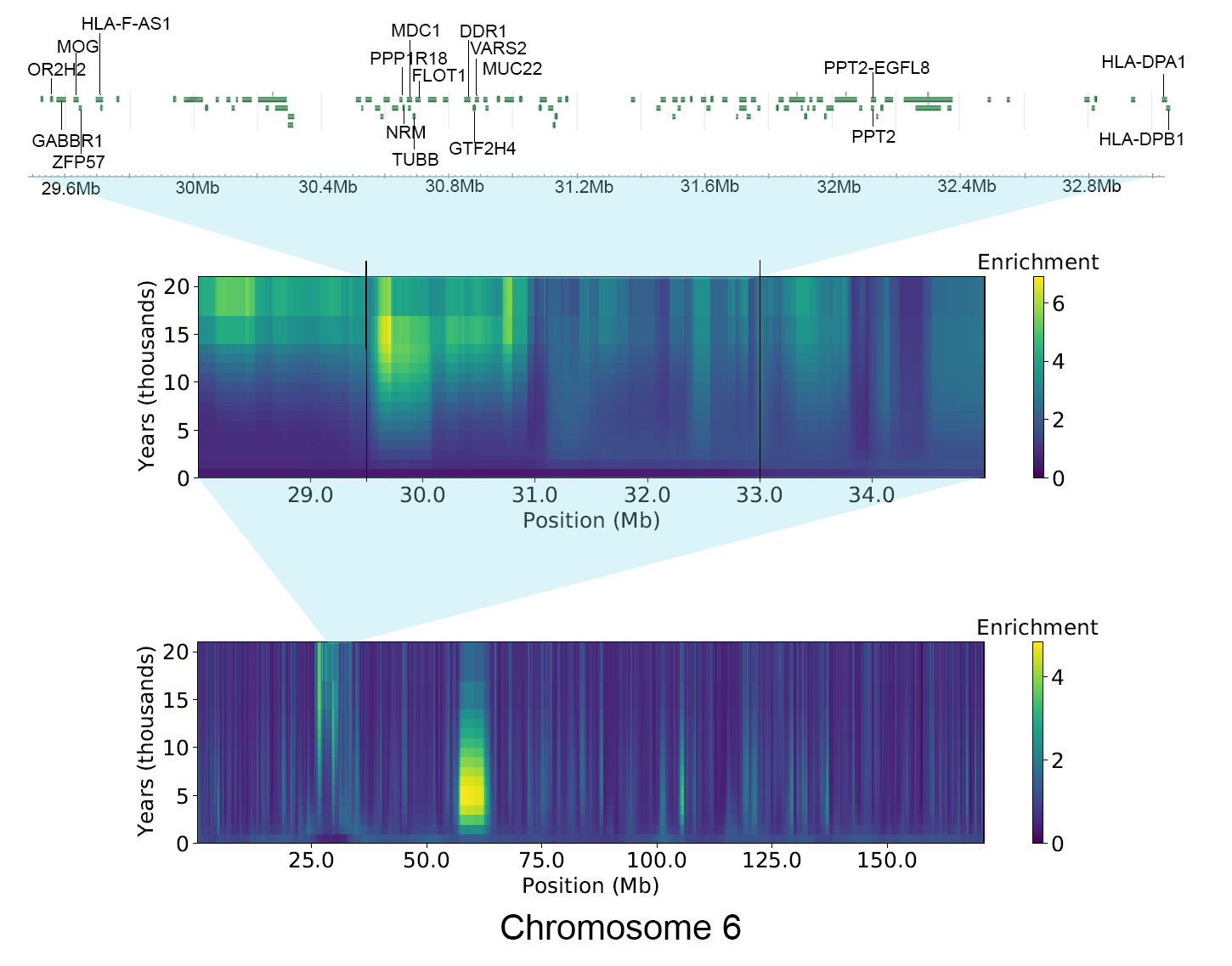

Supplement: S9 Fig — ASMC results for chromosome 6, showing a zoom in the region from 29.6MbMb to 32.8Mb, on the top we describe the genes located within this region. (TIF) [file pone.0271767.s009.tif]
